# A Survey Study of Patient‐Physician Communication and Treatment Decision‐Making Preference at Treatment Initiation and After Disease Stabilization in Newly Diagnosed Multiple Myeloma

**DOI:** 10.1002/jha2.70247

**Published:** 2026-04-26

**Authors:** Hitoshi Hanamoto, Masaki Iino, Kyoko Joko, Kaho Kidera, Takahiro Yoshida, Kiyoshi Okazuka, Hirohiko Shibayama

**Affiliations:** ^1^ Department of Hematology Kindai University Nara Hospital Ikoma Nara Japan; ^2^ Department of Hematology and Hematopoietic Stem Cell Transplantation Yamanashi Prefectural Central Hospital Yamanashi Japan; ^3^ Myeloma Patients and Families, Japan Osaka Japan; ^4^ Japan Medical Affairs Japan Oncology Business Unit Takeda Pharmaceutical Company Limited Tokyo Japan; ^5^ Department of Hematology National Hospital Organization Osaka National Hospital Osaka Japan

**Keywords:** communication, decision‐making, hematological malignancies, multiple myeloma, patient preference, patient‐centered care

## Abstract

**Introduction:**

We present real‐world evidence of patient‐physician communication for patients with newly diagnosed multiple myeloma (NDMM) who have not received hematopoietic stem cell transplantation.

**Methods:**

An observational survey study was conducted in Japan (September‒November 2024). Patients with NDMM completed a self‐reported 34‐item survey (online or paper‐based), and hematologists treating patients with multiple myeloma completed a self‐reported 18‐item survey online. Communication status between patients and physicians and information on patients' treatment expectations, values, emotions, knowledge, and treatment decision‐making preferences at both treatment initiation and disease stabilization were summarized.

**Results:**

Overall, 220 patients and 120 physicians were included. At both treatment initiation and disease stabilization, 45.9% and 50.3% of patients, respectively, had treatment options presented or explained to them, and 23.6% and 25.2%, respectively, were asked about their preferences; conversely, 82.5% and 65.0% of physicians, respectively, presented or explained treatment options to patients, and 67.5% and 50.8%, respectively, asked patients about their preferences. Patients' emotions shifted from negative to positive, their knowledge of the disease and treatment increased, and their treatment expectations changed from treatment initiation to disease stabilization. Overall, 44.5% of patients preferred a shared role in decision‐making; however, only 21.8% had a shared role in actual practice at treatment initiation.

**Conclusion:**

There was a discrepancy in perceptions of communication between patients and physicians. Patients' expectations, emotions, and knowledge changed from treatment initiation to disease stabilization. Physicians should understand these changes and communicate more effectively about treatment options and plans at both treatment initiation and disease stabilization.

**Trial Registration**: University Hospital Medical Information Network Center: UMIN000055606

## 1 Introduction

Survival of patients with multiple myeloma (MM) has been extended with the launch of many promising drugs in the past 20 years [1]; disease prognosis is improving, with deep response often achieved in > 50% of patients with newly diagnosed MM (NDMM) [2]. However, MM remains incurable, and many patients experience relapse as the disease progresses [3]. In clinical practice, MM treatment should account for not only the various treatment options and patient characteristics, but also patient preferences. Therefore, patient‐physician communication is crucial [4].

Patient‐centered communication (PCC) is a two‐way communication between patients and healthcare providers that encourages patients to actively engage in treatment; PCC can improve self‐efficacy and quality of life (QoL), thereby improving the physical and emotional well‐being of patients with cancer [5, 6, 7]. PCC comprises six core components: fostering healing relationships, exchanging information, responding to emotions, managing uncertainty, making decisions, and enabling patient self‐management [6]. With the American Society of Clinical Oncology publishing a consensus guideline on communication between patients and healthcare providers [8], PCC has recently attracted attention in the field of oncology [7].

Several oncology studies have reported on patient‐physician communication, including treatment decisions [9, 10, 11, 12, 13]. A survey of patients and physicians described treatment decision‐making, providing real‐world evidence of communication in relapsed/refractory MM (RRMM) [9]. In addition, a descriptive cross‐sectional study of older patients with symptomatic NDMM reported on patient‐physician communication, focusing on patients' preferences in treatment decision‐making at treatment initiation [10]. NDMM is associated with long‐term treatment, with current options offering improved disease control [1]; patients' emotions, disease knowledge, understanding, treatment expectations, values, and treatment decision‐making preferences are expected to change over the treatment course. Moreover, given the prolonged prognosis and increasing treatment complexity, understanding these changes is important to ensure patients' well‐being throughout the disease course. However, no studies have yet investigated these changes over time in patients with NDMM.

We aimed to explore and clarify patient‐physician communication for NDMM. Changes in patients' treatment expectations, values, emotions, knowledge, understanding, and treatment decision‐making preferences at treatment initiation and after disease stabilization were evaluated.

## 2 Methods

### 2.1 Study Design

This was an observational survey study of patients with NDMM who had not received hematopoietic stem cell transplantation and hematologists who treat patients with MM (study registration: UMIN000055606). The survey was conducted between September 26 and November 8, 2024, in accordance with the protocol, Declaration of Helsinki, and all applicable laws and regulations. The protocol was approved by the Medical Corporation TOUKEIKAI Kitamachi Clinic ethics review board (approval number: BGQ10450). Patients and physicians provided consent at survey registration. Data collected were de‐identified and anonymized.

The patient questionnaire (online/paper) comprised 34 questions on communication with physicians, treatment expectations, values, emotions, knowledge, and understanding of the disease and treatment options, and treatment decision‐making preferences at treatment initiation and disease stabilization. The physician questionnaire (online) comprised 18 questions on communication with patients, treatment expectations, and perception of patients' treatment decision‐making preferences at treatment initiation and disease stabilization. See Supplemental Methods for questionnaire development and data collection.

### 2.2 Participant Recruitment

Patients were recruited through a Japanese patient advocacy group for myeloma (Nihon Kotsuzuishu Kanja No Kai [Myeloma Patients and Families, Japan]). The questionnaire was mailed to 1431 patients registered in the patient advocacy group as of September 15, 2024. Eligible patients were aged ≥ 18 years, diagnosed with MM, treated with induction therapy without subsequent hematopoietic stem cell transplantation, under observation or had a stable clinical condition, and agreed to complete the survey.

Physicians were recruited through the m3.com platform (M3 Inc., Tokyo, Japan). Eligible physicians were hematologists who provided medical examination to ≥ 2 patients with MM per year and who agreed to complete the survey.

### 2.3 Statistical Analysis

The target sample sizes were 100 participants each (Supplemental Methods). For patients, all collected data were aggregated; for physicians, data from a maximum of 120 participants were included. All responses were summarized for patients who had answered the final question (online) or ≥ 1 of the attribute questions in the final section (paper). Proportions of patients with each level of knowledge and understanding, emotion, and value at treatment initiation and disease stabilization were calculated. As exploratory analyses, overall changes in patients' knowledge and understanding, and values, were analyzed using the Wilcoxon signed rank test and chi‐square test, respectively. The overall number of positive (optimistic/hopeful, excited, confident) and negative (worried, confused, fearful, sad, powerless, disappointed, apologetic, guilty) emotions was analyzed using the Wilcoxon signed rank test. *p*‐values of 0.05 indicated statistical significance. The terms “frustrated” and “sense of failure” were incorporated into the questionnaire for treatment initiation and disease stabilization, respectively; these were excluded from the analysis due to nuanced differences in their meanings. No or missing responses were defined as missing.

Statistical analyses were conducted using BellCurve Hideyoshi Dplus (Social Survey Research Information Co., Ltd., Tokyo, Japan), BellCurve for Excel (Social Survey Research Information Co., Ltd., Tokyo, Japan), and R (R Foundation for Statistical Computing, Vienna, Austria).

## 3 Results

### 3.1 Demographic and Baseline Clinical Characteristics

Of the 329 patients who answered the questionnaire, 220 were included after excluding those not meeting the eligibility criteria (*n = *98), those not consenting to participate (*n = *2), those with incomplete responses (*n = *8), and those with duplicated responses on paper and online (*n = *1). A total of 120 physicians who completed the questionnaire were included.

Median patient age was 73.5 years, and most patients were diagnosed with MM for ≥ 3 years. Common induction regimens were Bd (bortezomib, dexamethasone) (19.1%), DLd (daratumumab, lenalidomide, dexamethasone) (11.8%), and Ld (lenalidomide, dexamethasone) (11.4%). Distribution of healthcare facilities attended by patients and physicians' practice setting was similar (Table 1).

**TABLE 1:** Patient‐ and physician‐reported characteristics.

**Characteristic**	**Patients (*N *= 220)**	**Physicians (*N *= 120)**
Age, years		
Median (range)	73.5 (43‒89)^a^	—
≤ 55	19 (8.6)	—
> 55 to ≤ 65	39 (17.7)	—
> 65 to ≤ 75	76 (34.5)	—
> 75	78 (35.5)	—
Prefer not to answer/missing data	8 (3.6)	—
Sex		
Male	108 (49.1)	—
Female	105 (47.7)	—
Prefer not to answer/missing data	7 (3.2)	—
Time since diagnosis		
< 6 months	3 (1.4)	—
6 months to < 12 months	11 (5.0)	—
1 to < 2 years	16 (7.3)	—
2 to < 3 years	17 (7.7)	—
≥ 3 years	173 (78.6)	—
Prefer not to answer/missing data	0 (0)	—
Induction regimen^b^		
D‐MPB	22 (10.0)	—
DLd	26 (11.8)	—
Bd	42 (19.1)	—
Ld	25 (11.4)	—
Other	72 (32.7)	—
Do not remember	33 (15.0)	—
Prefer not to answer/missing data	0 (0)	—
Healthcare attended by patients/physician practice setting		
Cancer center/academia	89 (40.5)	43 (35.8)
Community hospitals (national/government hospitals, public hospitals, and general hospitals other than cancer center/academia), medical offices/clinics	131 (59.5)	76 (63.3)
Other	0 (0)	1 (0.8)
Prefer not to answer/missing data	0 (0)	—
Work status		
Currently working	53 (24.1)	—
Not working	161 (73.2)	—
Prefer not to answer/missing data	6 (2.7)	—
Cohabitation status		
Living with family	187 (85.0)	—
Living alone	30 (13.6)	—
Prefer not to answer/missing data	3 (1.4)	—
Place of residence/practice		
Hokkaido/Tohoku	12 (5.5)	20 (16.7)
Kanto	97 (44.1)	37 (30.8)
Chubu	32 (14.5)	21 (17.5)
Kinki	44 (20.0)	19 (15.8)
Chugoku/Shikoku	19 (8.6)	7 (5.8)
Kyushu/Okinawa	8 (3.6)	16 (13.3)
Prefer not to answer/missing data	8 (3.6)	0 (0)

*Note*: Data are *n* (%) unless otherwise indicated.

Abbreviations: Bd, bortezomib, dexamethasone; DLd, daratumumab, lenalidomide, dexamethasone; D‐MPB, daratumumab, melphalan, prednisolone, bortezomib; Ld, lenalidomide, dexamethasone.

^a^ *n *= 212.

^b^ Regimen abbreviations are per the Japanese guidelines.

### 3.2 Treatment Expectations

**FIGURE 1:**
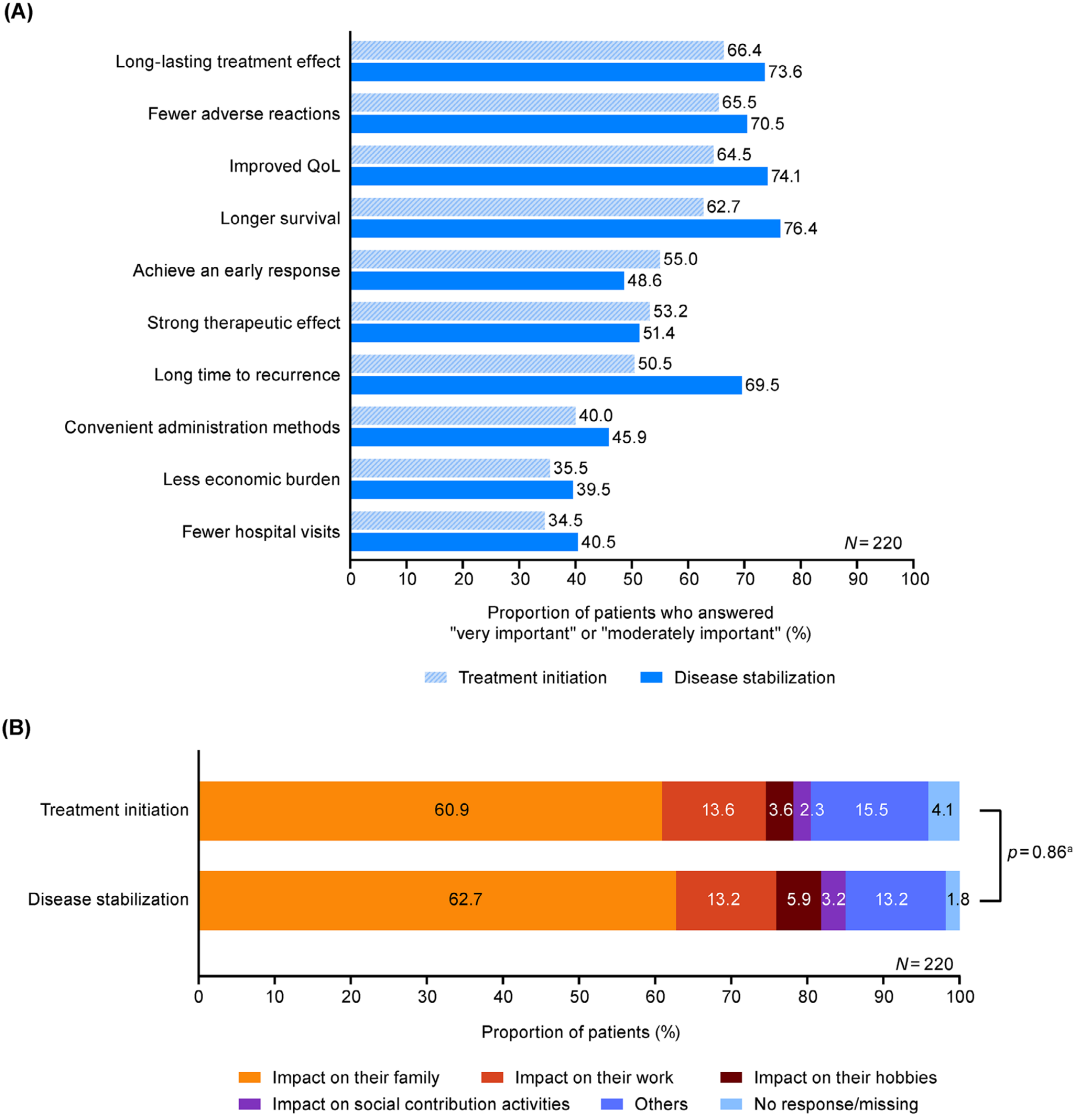
(A) Patients' treatment expectations, (B) patients' values influencing treatment expectations, at treatment initiation and disease stabilization. Patients rated the importance of each treatment attribute on a scale of "not important at all" to “very important”; the figure shows the proportion of patients who rated “very important” or “moderately important.” Patients also selected the values that influenced their rating of treatment attributes. ^a^ *p*‐value calculated using chi‐square test shows the overall difference in patients' values between treatment initiation and disease stabilization. QoL, quality of life.

Common treatment attributes that patients deemed important (“very important” or “moderately important”) were “long‐lasting treatment effect” (66.4%) and “fewer adverse reactions” (65.5%) at treatment initiation, and “longer survival” (76.4%) and “improved QoL” (74.1%) at disease stabilization (Figure 1A). The importance of each treatment attribute generally increased from treatment initiation to disease stabilization; the increase was particularly notable for “long time to recurrence” (Figure 1A). At both treatment phases, the most common value influencing patients' rating of treatment attributes was “impact on their family” (60.9%) (Figure 1B). No significant change in patients' values was observed between the treatment phases (*p = *0.86).

Physicians showed similar patterns to the patients in the treatment attributes they frequently deemed important (Figure S1).

### 3.3 Emotions, Knowledge, and Understanding of MM and Its Treatment

**FIGURE 2:**
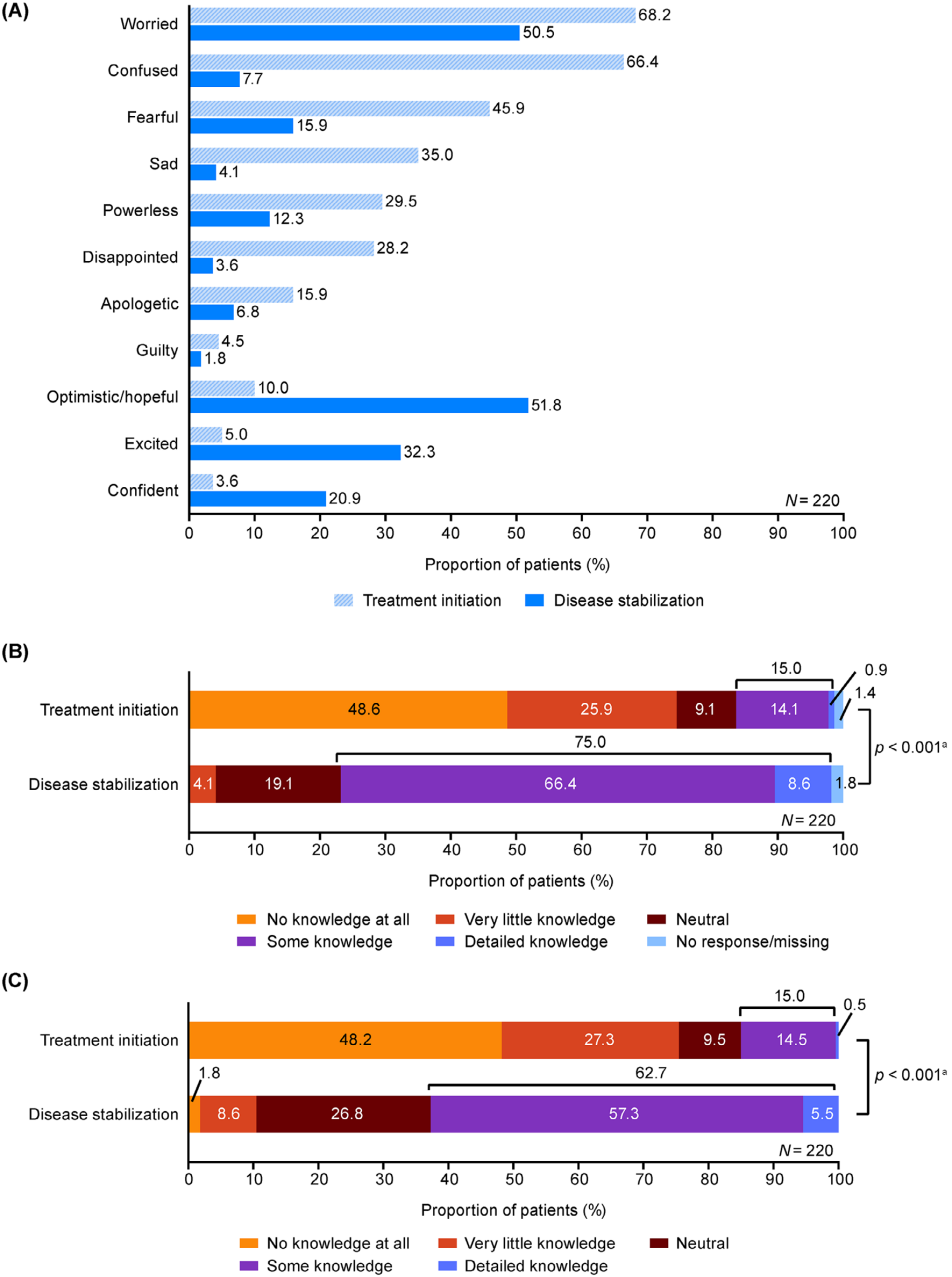
Patients' (A) emotions, (B) knowledge of the disease, (C) knowledge of the treatment options, at treatment initiation and disease stabilization. Patients answered questions about their emotions and level of knowledge. Multiple responses for emotions were allowed. ^a^ *p*‐values calculated by Wilcoxon signed rank test show the overall differences in patients' knowledge of the disease and treatment options between treatment initiation and disease stabilization.

At treatment initiation, the proportion of patients with negative emotions was high, and with positive emotions was low; the two most common emotions reported were “worried” (68.2%) and “confused” (66.4%) (Figure 2A). At disease stabilization, the proportions of patients with negative emotions decreased, but “worried” was still reported by 50.5% of patients. Overall, the number of negative emotions significantly decreased (*p *< 0.001), and the number of positive emotions significantly increased (*p *< 0.001), from treatment initiation to disease stabilization.

Patients' knowledge of the disease and treatment options (“some knowledge” and “detailed knowledge”) significantly increased from treatment initiation to disease stabilization (disease: 15.0% vs. 75.0%, treatment options: 15.0% vs. 62.7%, respectively; *p *< 0.001 for each) (Figure 2B,C). The proportion of patients who understood their physician's explanations about the disease and treatment options also generally increased from treatment initiation to disease stabilization (Figure S2). Patients often obtained information from physicians (85.9%) and a patient advocacy group (82.7%) (Figure S3).

### 3.4 Patient‐Physician Communication About Treatments

**FIGURE 3:**
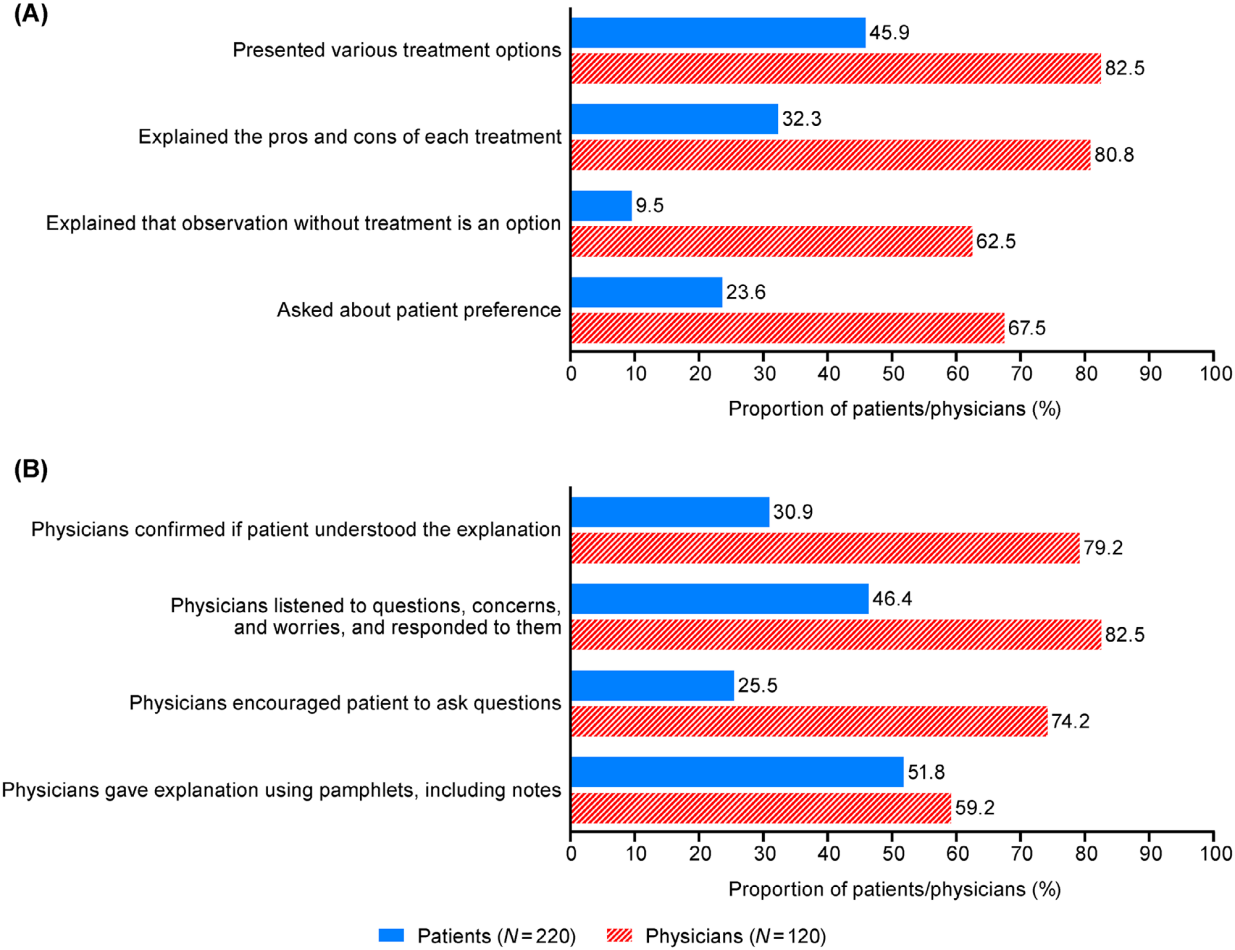
The status of patient‐physician communication at treatment initiation: (A) communication regarding treatment options, (B) how patients/physicians handled the communication. Patients answered about the explanations they received and how the physicians handled the communication. Physicians answered about the explanations they provided and how they handled the communication.

Approximately half of physicians spent 15 to < 30 min on communication at treatment initiation, but only 25.8% at disease stabilization (Table S1). At treatment initiation, 45.9% and 23.6% of patients reported being presented with various treatment options and being asked about their treatment preferences, respectively (Figure 3A). Conversely, 82.5% and 67.5% of physicians reported having presented various treatment options and asked patients about their preferences, respectively (Figure 3A). Moreover, 30.9% and 25.5% of patients answered that their understanding was confirmed by the physician and they were encouraged to ask questions, respectively (Figure 3B). However, the proportion of physicians who said they confirmed the patient's understanding was 79.2%, and those who said they encouraged the patient to ask questions was 74.2%.

**FIGURE 4:**
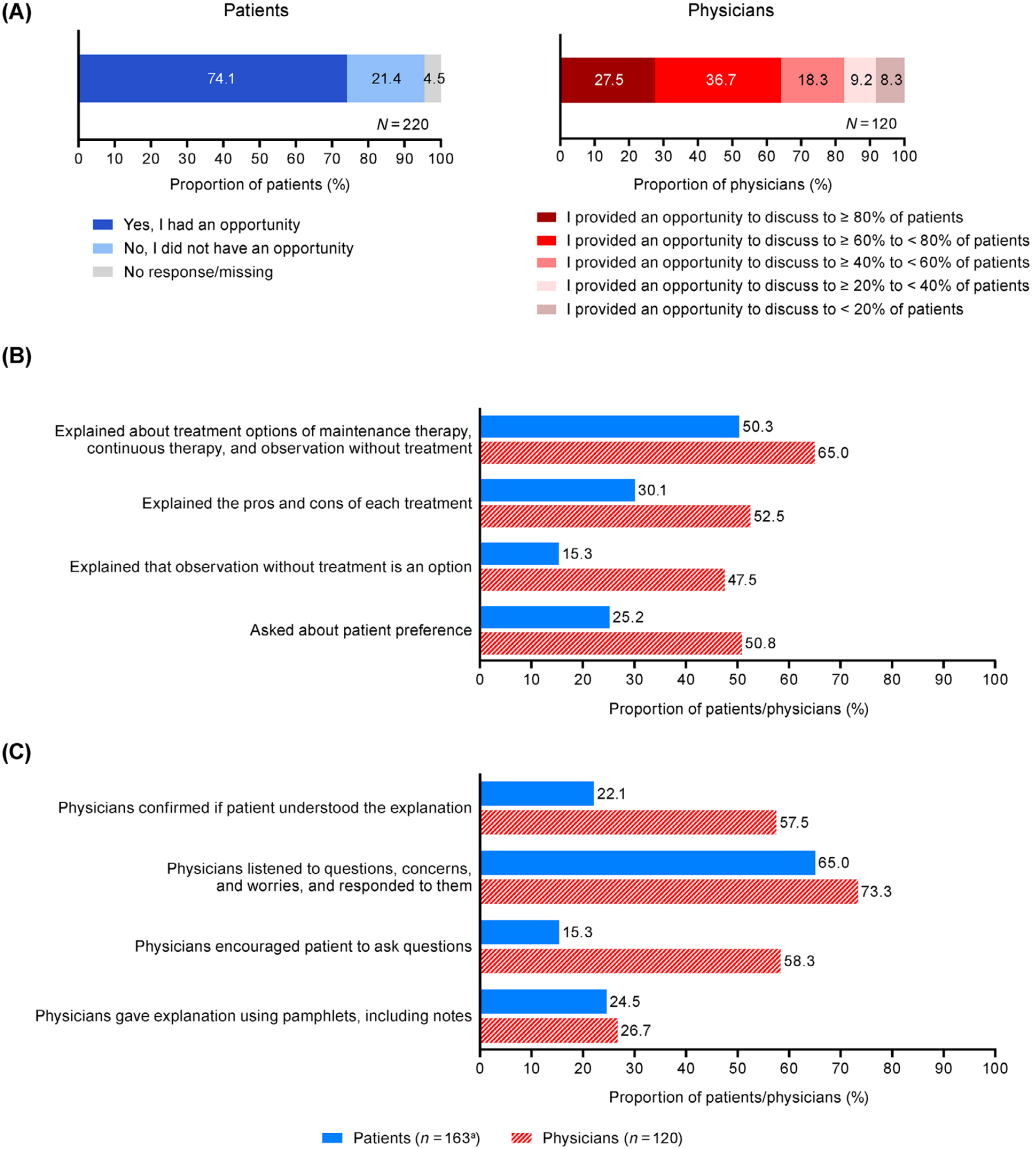
The status of patient‐physician communication at disease stabilization: (A) opportunity to discuss prospects and treatment strategies at disease stabilization, (B) communication regarding treatment options, (C) how patients/physicians handled the communication. Patients answered about the explanations they received and how the physicians handled the communication. Physicians answered about the explanations they provided and how they handled the communication. ^a^Patients who responded that they had an opportunity to discuss prospects and treatment strategies at disease stabilization.

At disease stabilization, 74.1% of patients answered that they had the opportunity to discuss treatment strategies and prospects, and 21.4% reported no opportunity; for physicians, 64.2% reported that they provided opportunities to discuss treatment strategies and prospects to ≥ 60% of patients (Figure 4A). The most common reason for physicians not to provide such opportunities was “patient's condition was stable” (62.5%) (Table S2). Of the 163 patients who reported having the opportunity for discussion, 50.3% answered that the treatment options were explained, and 25.2% answered that they were asked about their treatment preferences (Figure 4B). Conversely, 65.0% of physicians reported having explained the treatment options, and 50.8% reported asking the patients about their preferences (Figure 4B). Moreover, only 22.1% and 15.3% of patients answered that their understanding was confirmed by the physician and they were encouraged to ask questions, respectively; however, 57.5% and 58.3% of physicians answered that they confirmed the patient's understanding and encouraged the patient to ask questions, respectively (Figure 4C). The most common topic that patients hoped to discuss with their physicians was “duration of treatment” (63.2%) (Table S3).

### 3.5 Treatment Decision‐Making Preferences

**FIGURE 5:**
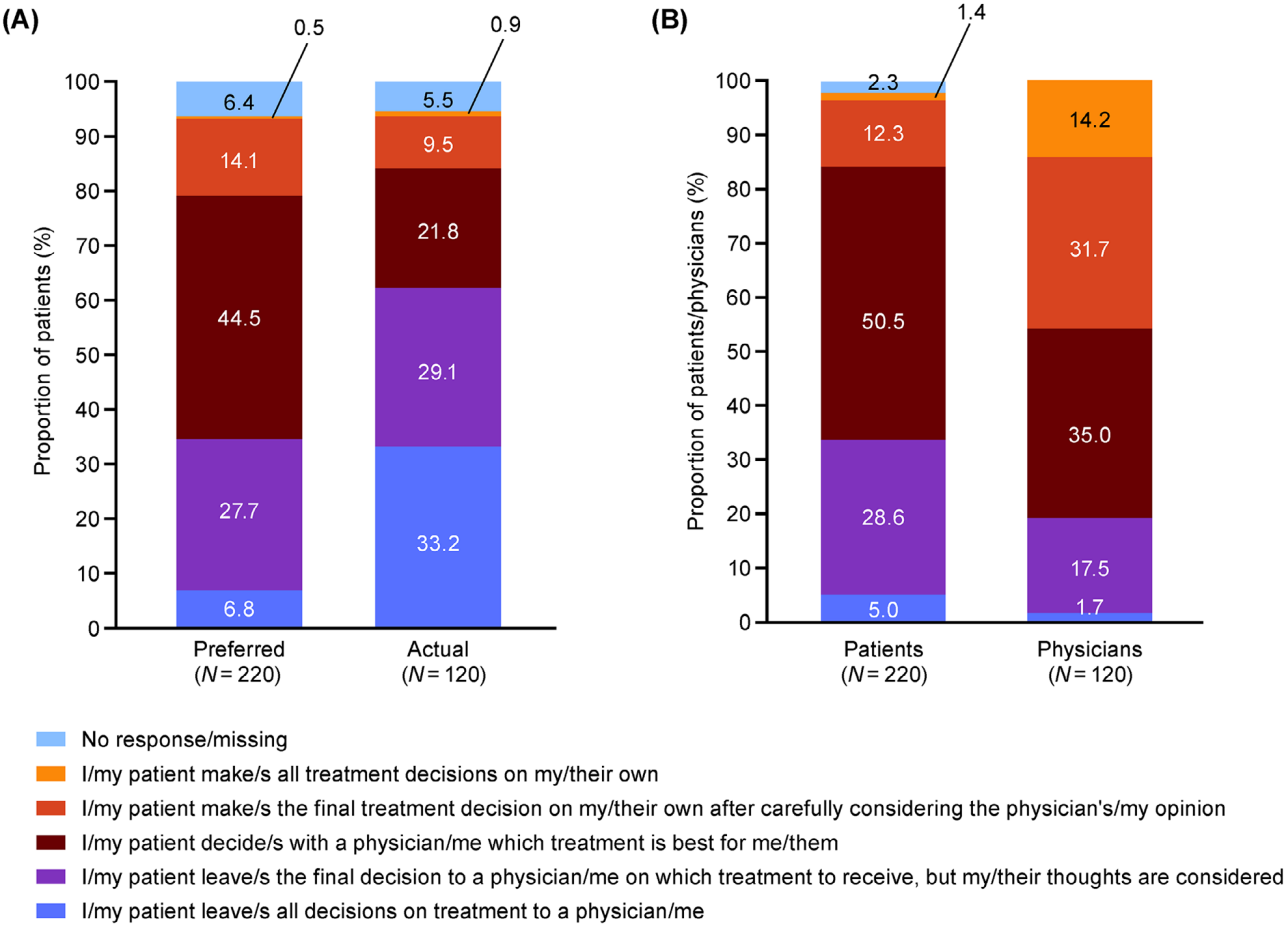
Treatment decision‐making approach: (A) patient's preferred role versus actual role at treatment initiation, (B) patient's preferred role versus physician's perspective on the patient's preferred role at disease stabilization. Patients answered about their preferred role and actual role at treatment initiation and their preferred role at disease stabilization. Physicians answered about their perception of the patient's preferred role at disease stabilization.

At treatment initiation, the preferred treatment decision‐making approaches reported by patients were “I decide with a physician which treatment is best for me” (44.5%) and “I leave the final decision to a physician on which treatment to receive, but my thoughts are considered” (27.7%) (Figure 5A). In reality, the two most common treatment decision‐making approaches taken in practice were “I leave all decisions on treatment to a physician” (33.2%) and “I leave the final decision to a physician on which treatment to receive, but my thoughts are considered” (29.1%) (Figure 5A). Similar to at treatment initiation, the preferred treatment decision‐making approach reported by patients at disease stabilization was “I decide with a physician which treatment is best for me” (50.5%) and “I leave the final decision to a physician on which treatment to receive, but my thoughts are considered” (28.6%) (Figure 5B). These responses differed from physicians' perceptions at disease stabilization; physicians selected “My patient decides with me which treatment is best for them” (35.0%) and “My patient makes the final treatment decision on their own after carefully considering my opinion” (31.7%) as the two most common approaches they believe the patients preferred (Figure 5B; Figure S4).

## 4 Discussion

This observational study explored patient‐physician communication among patients with NDMM and changes in patients' expectations, values, emotions, knowledge, and treatment decision‐making preferences from treatment initiation to disease stabilization. Patients indicated that communication on treatment options and preferences was insufficient at both treatment initiation and disease stabilization, but physicians indicated the opposite, suggesting different perceptions of patient‐physician communication. Patients' expectations, emotions, and disease knowledge and understanding changed between treatment initiation and disease stabilization. Almost half of the patients preferred to share treatment decisions with physicians at both treatment initiation and disease stabilization; however, the actual decision‐making approach differed at treatment initiation.

In this study, physicians made time for communication during the medical consultation at both treatment phases. At disease stabilization, although 65.0% of physicians indicated that they provided explanations about treatment options, only 50.3% of patients reported receiving such explanations. These results suggest that patients possibly did not receive adequate explanations from physicians about treatment options, and that perceptions of patient‐physician communication differ. The Japanese clinical practice guidelines for hematological malignancies list DLd and D‐MPB (daratumumab, melphalan, prednisolone, bortezomib) as the recommended standard‐of‐care regimen for patients with transplant‐ineligible MM; other treatments include Ld, MPB (melphalan, prednisolone, bortezomib), Bd, and modified BLd (bortezomib, lenalidomide, dexamethasone) regimens [14]. Treatment options after achieving a response include continuing DLd or transitioning from D‐MPB to daratumumab continuous therapy, lenalidomide maintenance therapy, ixazomib maintenance therapy, or observation without treatment. Given the availability of various treatments and the fact that patients' treatment expectations appear to change as their treatment progresses, physicians need to actively communicate with patients, including about subsequent treatment options, even at disease stabilization.

This study reported that 21.4% of patients had no opportunity to discuss their prospects or treatment strategies with their physicians when their condition stabilized, suggesting that patient‐physician communication was not sufficient at disease stabilization for some patients. Consistent with previous findings [15], many patients wanted explanations on future treatment strategies (e.g., duration of treatment), indicating that patients need the opportunity to discuss prospects and future treatment strategies with their physicians again at disease stabilization. However, physicians may not provide such opportunities if the patient's condition is stable. This highlights another discrepancy between physicians' perspectives and patients' needs.

Emotions and physical symptoms are interrelated [16], and psychological changes are thought to occur as myeloma‐specific symptoms improve with treatment. In a case‐control study conducted in patients with breast cancer, the proportion of patients with more positive versus negative emotions was higher during the post‐treatment rehabilitation period than during the treatment period [17]. Similarly, in the current study, patients' negative emotions decreased and positive emotions increased from treatment initiation to disease stabilization, revealing changes in patients' emotions over time. As MM prognosis has improved in recent years, similar to breast cancer, experiencing symptom relief or perceiving progress toward a potential cure may contribute to enhancing positive emotions in patients with MM. Of note, approximately half of patients in this study continued to feel “worried” even at disease stabilization, consistent with the results of a previous study reporting a relatively stable prevalence of anxiety in the first 2 years after MM diagnosis [18]. Patients with MM repeatedly experience relapse and remission; therefore, they may always be anxious about the next relapse even when their condition is stable. Studies have shown that PCC, including asking questions, expressing empathy, and making efforts to understand the patient during medical consultations, can help reduce anxiety [19, 20]. Therefore, it is important for physicians to consider the patient's emotional improvement and ongoing concerns in their communication.

In this study, patients' knowledge of the disease and treatment options increased from treatment initiation to disease stabilization. Patients appeared to have obtained information from various channels, such as from physicians and patient advocacy groups. Our results suggest that patients' understanding of physicians' explanations also increased as their knowledge increased.

Unlike patients with lung cancer who prioritize longer survival and are willing to accept side effects and reduced QoL [12], in our study the importance of most treatment attributes increased from treatment initiation to disease stabilization. This may be characteristic of MM because patients live with the disease for a long time and hope to continue their normal daily lives during treatment [21]. Therefore, patients with MM would have greater expectations as they return to “normal life.” This was also supported by the fact that most patients deem the impact on their family and work as important considerations affecting their views on treatment expectations. Of note, the proportion of patients who answered “long time to recurrence” as a treatment expectation increased by approximately 20% from treatment initiation to disease stabilization, perhaps because patients realized that MM is a recurrent disease. Moreover, similar to the results of the survey study of RRMM [9], patients' treatment expectations in this study were generally aligned with those of the physicians.

Patients with solid tumors have been reported to prefer a shared decision‐making role [22], while patients with hematological malignancies prefer leaving the treatment decisions to their physicians [23, 24], possibly due to the urgency and complexity of the therapies [24]. However, studies reported that many patients with RRMM or NDMM preferred to make treatment decisions with their physicians [9, 10]. Consistent results were observed in the current study, with approximately half of the patients reporting that they prefer to make decisions with their physicians at both treatment initiation and disease stabilization. These results suggest that patients with MM have a greater desire to participate in the treatment decision‐making process. However, in reality, at treatment initiation, one‐third of patients had left all treatment decisions to their physician, with only one‐fifth of patients making decisions with a physician. These results were similar to previous studies reporting a difference in the preferred versus perceived role among patients with solid tumors and hematological malignancies [11, 25], with the conclusion that patients' preferences should be considered more by physicians during treatment decision‐making. Moreover, in the current study, physicians' perceptions of the patient's preferred treatment decision‐making approach and the patient's actual preferences differed. Patients are reported to have fewer treatment regrets and greater satisfaction and improved QoL when their preferred role in treatment decision‐making aligns with their actual role [26, 27]. Effectively eliciting information about each patient's preferred role in treatment decision‐making and tailoring communication accordingly may enhance the patient's satisfaction, QoL, and well‐being [5, 6, 7].

The generalizability of the study results may be limited because this study only included patients and physicians in Japan. Furthermore, patients were recruited from an advocacy group and had stable disease; therefore, they were more likely to be satisfied with their treatment and may have had higher health literacy than the general patient population. Bias relating to experience, understanding, and treatment expectations may also exist as patients with a relatively short duration since diagnosis were included. In addition, the questionnaire was not validated, and the responses are subject to recall bias. Importantly, patient and physician responses were not paired; therefore, a simple comparison requires caution.

## 5 Conclusions

This study revealed some notable discrepancies in perceptions of patient‐physician communication and treatment decision‐making preferences between patients with NDMM and the physicians and demonstrated changes in patients' treatment expectations, emotions, knowledge, and understanding from treatment initiation to disease stabilization. Both patients and physicians need to recognize discrepancies in their perceptions; physicians also need to recognize changes in patients and communicate more effectively about treatment options and future treatment plans at each treatment phase. Discussing treatment plans at disease stabilization is essential from a patient‐centered care perspective, with consideration given to reduced‐intensity or maintenance therapies and the patient's evolving knowledge and psychological state. Further studies and discussion are needed to explore effective patient‐physician communication and enhance the well‐being of patients with MM.

## Author Contributions

All authors were investigators, were involved in the study design and data collection, and participated in the interpretation of study results and in the drafting, critical revision, and approval of the final version of the manuscript. K.K., T.Y., and K.O. conducted the statistical analysis.

## Funding

This study was funded by Takeda Pharmaceutical Company Limited, which was involved in the study design, data collection, data analysis, and preparation of the manuscript.

## Ethics Statement

This study was conducted in accordance with the protocol, the Declaration of Helsinki, and all applicable laws and regulations, and the protocol was approved by the ethics review board.

## Consent

Patients and physicians provide consent at the time of survey registration. Data collected during the study were de‐identified and anonymized.

## Conflicts of Interest

Hitoshi Hanamoto has received honoraria from CSL Behring K.K., Janssen Pharmaceutical K.K., Pfizer Japan, and Takeda Pharmaceutical Company Limited. Masaki Iino has no conflict of interest to declare. Kyoko Joko has received consulting fees from Takeda Pharmaceutical Company Limited. Kaho Kidera, Takahiro Yoshida, and Kiyoshi Okazuka are employees of Takeda Pharmaceutical Company Limited, and Kaho Kidera owns stock via the employee ownership program. Hirohiko Shibayama has received honoraria from AstraZeneca K.K., Chugai Pharmaceutical Co., Ltd., Meiji Seika Pharma Co., Ltd., Nippon Shinyaku Co., Ltd., and Ono Pharmaceutical Co., Ltd, and has participated in data safety monitoring or advisory boards for Chugai Pharmaceutical Co., Ltd. and Fujimoto Pharmaceutical Corporation.

## Supporting information




**Supporting File 1**: jha270247‐sup‐0001‐SuppMat.docx

## Data Availability

The datasets generated during and/or analyzed during the current study are available from the corresponding author on reasonable request.
