# Supplementary material for: A Survey Study of Patient‐Physician Communication and Treatment Decision‐Making Preference at Treatment Initiation and After Disease Stabilization in Newly Diagnosed Multiple Myeloma
Source: EJHaem. 2026 Apr 26;7(2):e70247. doi: 10.1002/jha2.70247 (PMC13110793; doi:10.1002/jha2.70247)

SUPPORTING INFORMATION

**A Survey Study of Patient-Physician Communication and Treatment Decision-Making Preference at Treatment Initiation and After Disease Stabilization in Newly Diagnosed Multiple Myeloma**

Hitoshi Hanamoto^1^, Masaki Iino^2^, Kyoko Joko^3^, Kaho Kidera^4^, Takahiro Yoshida^4^, Kiyoshi Okazuka^4^, Hirohiko Shibayama^5^

**AUTHORS’ AFFILIATIONS**

^1^Department of Hematology, Kindai University Nara Hospital, Ikoma, Nara, Japan

^2^Department of Hematology and Hematopoietic Stem Cell Transplantation, Yamanashi Prefectural Central Hospital, Yamanashi, Japan

^3^Myeloma Patients and Families, Japan, Osaka, Japan

^4^Japan Medical Affairs, Japan Oncology Business Unit, Takeda Pharmaceutical Company Limited, Tokyo, Japan

^5^Department of Hematology, National Hospital Organization Osaka National Hospital, Osaka, Japan

**CORRESPONDING AUTHOR**

Hirohiko Shibayama

Department of Hematology, National Hospital Organization Osaka National Hospital

2-1-14, Hoenzaka, Chuo-ku, Osaka-shi

Osaka, 540-0006, Japan

Email: shibayama.hirohiko.ec@mail.hosp.go.jp

**LIST OF TABLES AND FIGURES**

Supplemental Methods

**Supplemental Text 1 |** English translation of the patient questionnaire.

**Supplemental Text 2 |** English translation of the physician questionnaire.

**Table S1** | Time spent during medical consultation reported by patients and physicians at treatment initiation and disease stabilization.

**Table S2** | Reasons why physicians did not provide opportunities to discuss prospects and treatment strategies at disease stabilization.

**Table S3** | Topics that patients hoped to discuss with the physician at disease stabilization.

**Figure S1** | Physicians’ treatment expectations at treatment initiation and disease stabilization.

**Figure S2** | Patients’ level of understanding of **(A)** the disease, **(B)** treatment options at treatment initiation and disease stabilization.

**Figure S3** | Patients’ sources of information on the disease and treatment.

**Figure S4** | Physicians’ perceptions: patients’ preferred treatment decision-making role at treatment initiation and disease stabilization.

Supplemental Methods

Questionnaire Development and Data Collection

The questionnaires were developed by a research steering committee comprising MM specialists and the representative of the patient advocacy group based on the results of previous studies [1, 2] and a qualitative interview conducted with four patients with MM prior to this study. A patient advocacy group representative reviewed the questionnaire to confirm that the content and wording were easily understandable for patients. The questionnaires were written in Japanese (see Supplemental Texts 1 and 2 for the English translations).

Patients completed the survey (online or paper-based) free of charge. Paper-based surveys were mailed to the patients from the Social Survey Research Information Co., Ltd. (SSRI; Tokyo, Japan); online surveys were completed through the SSRI website. Physicians completed the survey online through the SSRI website; those who completed the survey in accordance with the m3.com terms and conditions received points that could be exchanged for gifts and other types of monetary points.

Statistical Analysis

The sample size was determined by estimating the number of eligible patients based on the results of a survey conducted in 2022 by the patient advocacy group (unpublished data) for patients, and based on the number of hematologists registered in the m3.com platform, accounting for the response rates of a general survey for physicians.

References

[1] Bylund CL, Eggly S, LeBlanc TW, et al. Survey of patients and physicians on shared decision-making in treatment selection in relapsed/refractory multiple myeloma. Transl Behav Med. 2023;13(4):255–67.

[2] Tariman JD, Doorenbos A, Schepp KG, Singhal S, Berry DL. Older adults newly diagnosed with symptomatic myeloma and treatment decision making. Oncol Nurs Forum. 2014;41(4):411–9.

**Supplemental Text 1 |** English translation of the patient questionnaire.

**Patient Questionnaire**

**Please return the questionnaire to us by Wednesday, November 6, 2024.**

**Real-World Survey of Patients With Multiple Myeloma**


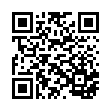


- **You can also respond to this survey online via the URL or QR code below.**
- **Please respond using a PC, smartphone, or tablet.**
- **Please do not respond via both mail and the internet.**

**https://www.ssri.co.jp/s/74h5hxty/3525279920/**

[Sponsor] Takeda Pharmaceutical Company Limited

[Research company] Social Survey Research Information Co., Ltd.

**If you would like to continue completing this questionnaire, please check the following eligibility criteria.**

**Please place a check mark in the box that corresponds to you.**

□ **I am 18 years or older.**

**□ I was diagnosed with multiple myeloma by a physician.
I have received my first treatment and am currently in a stable condition.**

**□ I have never had a transplant.**

**In this context, “Currently stable following initial treatment” refers**

**to situations where the initial treatment relieves symptoms, improves test values, or when the physician states, “The treatment is working” or “The disease has stabilized.”**

**Patients continuing initial treatment, those who have completed initial treatment and are under observation, and those who are on maintenance therapy following their initial treatment are all included within the scope of this survey.**

**If you have checked all the boxes, please turn the page and review the informed consent form.**

**If you have not checked all the boxes, you are not eligible to participate in this questionnaire.
Please discard this questionnaire.**

[Sponsor] Takeda Pharmaceutical Company Limited

[Research company] Social Survey Research Information Co., Ltd.

**Real-World Survey of Patients With Multiple Myeloma**

The survey will include a written letter of invitation and questions, and will take approximately 20 minutes to complete.

**[Informed Consent Form]**

If you understand the following precautions and would like to cooperate with the survey, please select “I agree” below.

- Purpose of this survey: This survey aims to assess the current state of and opinions on treatment selection among patients with multiple myeloma.
- Please answer each question by selecting the option that best describes your situation. Some of the questions may ask you to enter a number.
- Your participation in this survey is not mandatory. Your decision to answer is voluntary.
- If you wish to stop answering midway, you may do so immediately.
- If you choose not to answer or stop midway, you will not be disadvantaged in any way. The treatment you are currently receiving will not be affected, and your responses will not be shared with your regular physician.
- Answers remain confidential throughout processing, storage, and disposal. Your personal information will not be disclosed.
- The results of this survey will be used solely for research purposes, processed statistically to ensure anonymity, and presented at academic conferences or published as a scholarly paper. The results will not be published in a way that reveals individual identities.
- This survey is being conducted by Social Survey Research Information Co., Ltd. at the request of the sponsor. All costs related to the survey will be paid for by the sponsor. The ethics committee has reviewed and confirmed in advance that no conflict of interest exists between the sponsor and the research company that could compromise the reliability of the survey, and that the appropriate implementation structure is in place.
- Intellectual property rights arising from this survey shall belong to the sponsor.
- The results of the survey may be provided to regulatory authorities and ethics committees as necessary, but individuals will remain anonymous in all cases.
- **If you decide to participate in the survey after reviewing the precautions on the left, please be sure to select “I agree” below before completing the questionnaire.**
- **If consent is not given and subsequent questions are answered,**

**the questionnaire will be treated as if “I agree” had been selected.**

**□ I agree □ I do not agree**

If you agree, please proceed to the next page.

**We would now like to ask about your situation at treatment initiation following your multiple myeloma diagnosis.**

How long ago were you diagnosed with multiple myeloma?

(Select one)

| **1.** | Less than 6 months |
| --- | --- |
| **2.** | 6 months or more to less than 1 year |
| **3.** | 1 year or more to less than 2 years |
| **4.** | 2 years or more to less than 3 years |
| **5.** | 3 years or more |

Please indicate the type of hospital **at treatment initiation**.

(Select one)

| **1.** | Cancer Center/University Hospital |
| --- | --- |
| **2.** | Community hospital (national/government hospital, public hospitals or general hospital other than the above), Medical office/Clinic |
| **3.** | Other |
| **4.** | I don’t know |

What was **the first treatment you received following your multiple myeloma diagnosis**?

(Select one)

| **1.** | D-MPB (daratumumab, melphalan, prednisolone, bortezomib) |
| --- | --- |
| **2.** | DLd (daratumumab, lenalidomide, dexamethasone) |
| **3.** | Bd (bortezomib, dexamethasone) |
| **4.** | Ld (lenalidomide, dexamethasone) |
| **5.** | Other |
| **6.** | I don’t remember |

**About consultations**

**During the period between your multiple myeloma diagnosis and treatment initiation**, was the environment conducive to listening to the physician’s explanation or asking questions?

(Select one)

| **1.** | Very conducive |
| --- | --- |
| **2.** | Moderately conducive |
| **3.** | Not sure |
| **4.** | Not very conducive |
| **5.** | Not conducive at all |


**Regarding the period between your multiple myeloma diagnosis and treatment initiation,**

How much **time has your physician spent explaining multiple myeloma (the disease and treatment options)** to you?

How much **time have you had to ask your physician questions or discuss your concerns**?

*If explanations and opportunities for questions occur multiple times, please provide the total time spent.

|  | **Amount of time spent listening to the physician’s explanation**  (Select one) | **Amount of time you were able to consult**  **with the physician and ask questions**  (Select one) |
| --- | --- | --- |
| Less than 15 minutes | **1** | **1** |
| 15 minutes to less than 30 minutes | **2** | **2** |
| 30 minutes to less than 1 hour | **3** | **3** |
| 1 hour or more | **4** | **4** |

Regarding the physician’s explanation, etc. **during the period between your multiple myeloma diagnosis and treatment** **initiation**…

(Select all that apply)

|  | **About the disease** |
| --- | --- |
| **1.** | The physician explained multiple myeloma |
| **2.** | The physician explained why treatment is necessary |
|  | **About the treatment options** |
| **3.** | The physician presented several treatment options |
| **4.** | The physician explained the pros and cons of each treatment |
| **5.** | The physician also presented observation without treatment as an option |
| **6.** | The physician asked about my preference |
|  | **About your prospects after treatment initiation** |
| **7.** | The physician explained my prospects after treatment initiation (duration of treatment, timing and methods used to assess treatment effectiveness, etc.) |
| **8.** | The physician informed me that treatment may be changed depending on the therapeutic effect and that I will be consulted at that time |
|  | **When you receive an explanation from the physician** |
| **9.** | The physician confirmed that I understood their explanation |
| **10.** | The physician listened to my questions, concerns, and worries and responded to them |
| **11.** | The physician encouraged me to ask questions |
| **12.** | The physician used pamphlets, including notes, to explain the information (and gave me a pamphlet) |
|  |  |
| **13.** | None of the above/I don’t remember |

How well did you understand **the explanation** given by the physician **about multiple myeloma** **during the period between** **diagnosis and treatment initiation?**

(Select one)

| Very well | Moderately well | Not sure | Not very well | Not at all |
| --- | --- | --- | --- | --- |
| **5** | **4** | **3** | **2** | **1** |

How well did you understand **the explanation** given by the physician **about treatment options** **during the period between diagnosis and treatment initiation**?

(Select one)

| Very well | Moderately well | Not sure | Not very well | Not at all |
| --- | --- | --- | --- | --- |
| **5** | **4** | **3** | **2** | **1** |

**Regarding your thoughts on treatment**

**At treatment initiation,** what were **your expectations for multiple myeloma treatment (treatment regimen)?** Please indicate the **degree of importance** you place on your expectations for the treatment (treatment regimen) you receive. Please also provide your **top five priorities**.

|  | Very important | Moderately important | Not sure | Not very important | Not important at all |  | Top five priorities |
| --- | --- | --- | --- | --- | --- | --- | --- |
|  | (Select one for each row) | | | | |  | (Enter number) |
| Longer survival | **5** | **4** | **3** | **2** | **1** |  | position |
| Long time to recurrence | **5** | **4** | **3** | **2** | **1** |  | position |
| Improved quality of life | **5** | **4** | **3** | **2** | **1** |  | position |
| Long-lasting treatment effect | **5** | **4** | **3** | **2** | **1** |  | position |
| Achieve an early response | **5** | **4** | **3** | **2** | **1** |  | position |
| Strong therapeutic effect | **5** | **4** | **3** | **2** | **1** |  | position |
| Fewer adverse reactions | **5** | **4** | **3** | **2** | **1** |  | position |
| Convenient administration methods (intravenous, subcutaneous, oral administration) | **5** | **4** | **3** | **2** | **1** |  | position |
| Fewer hospital visits | **5** | **4** | **3** | **2** | **1** |  | position |
| Less economic burden | **5** | **4** | **3** | **2** | **1** |  | position |

**At treatment initiation**, what was your priority (value) when considering your expectations for multiple myeloma treatment that you mentioned in your answer to Q8 on the previous page? Please select **the most appropriate option:**

(Select one)

| **1.** | Impact on my family |
| --- | --- |
| **2.** | Impact on my work |
| **3.** | Impact on my hobbies |
| **4.** | Impact on social engagement activities |
| **5.** | None of the above |


**At treatment initiation**, were you able to share with your physician the treatment expectations and priorities (values) that you mentioned in your answers to Q8 and Q9 above?

(Select one)

| **1.** | **I was able to share** my treatment expectations and priorities with my physician |
| --- | --- |
| **2.** | **I was not able to share** my treatment expectations and priorities with my physician |

**At treatment initiation**, how did you want to decide your treatment? Please describe your **preferred decision-making process**.

Also, how did you actually decide on your treatment with your physician? Please describe **how this was decided in a real-world setting**.

|  | **Ideal decision making**  (Select one) | **Real-world decision making**  (Select one) |
| --- | --- | --- |
| Make all treatment decisions  on my own | **1** | **1** |
| Make the final treatment decision on my own after carefully considering the physician’s opinion | **2** | **2** |
| Decide with the physician which treatment is best for me | **3** | **3** |
| Leave the final decision to the physician on which treatment to receive, but my thoughts are considered | **4** | **4** |
| Leave all decisions on treatment to the physician | **5** | **5** |

**We would now like to ask you about your current situation in which your condition is stable.**

**In this context, “Currently stable following initial treatment” refers to situations where the initial treatment relieves symptoms, improves test values, or when the physician states, “The treatment is working” or “The disease has stabilized.”**

**Patients continuing initial treatment, those who have completed initial treatment and are under observation, and those who are on maintenance therapy following their initial treatment are all included within the scope of this survey.**

Between treatment initiation and now, while your condition remains stable, have you been transferred from the hospital where you began treatment for multiple myeloma at any point?

(Select one)

| **1.** | No, I have not been transferred **→** proceed to **‎Q14** on the next page. |
| --- | --- |
| **2.** | Yes, I have been transferred to another hospital. |

**If you have been transferred to another hospital, please answer** [**Q13**](#link_Q13) **below.**


Please describe the type of hospital you transferred to (the hospital you are currently visiting).

(Select one)

| **1.** | Cancer Center/University Hospital |
| --- | --- |
| **2.** | Community hospital (national/government hospital, public hospital or general hospital other than the above), Medical office/Clinic |
| **3.** | Other |
| **4.** | I don’t know |

**↓**

**After answering, please proceed to ‎Q14 on the next page.**

**Regarding consultations**

**From this point forward, everyone will respond.**


**At present, with your condition stable**, are you in an environment that is conducive to listening to the physician’s explanations or asking questions?

(Select one)

| **1.** | The environment and atmosphere are very conducive |
| --- | --- |
| **2.** | The environment and atmosphere are moderately conducive |
| **3.** | Not sure |
| **4.** | The environment and atmosphere are not very conducive |
| **5.** | The environment and atmosphere are not conducive at all |


**At present, with your condition stable**, what is the average duration of your regular medical consultations?

(Select one)

| **1.** | Less than 5 minutes |
| --- | --- |
| **2.** | 5 minutes to less than 10 minutes |
| **3.** | 10 minutes to less than 20 minutes |
| **4.** | 20 minutes to less than 30 minutes |
| **5.** | 30 minutes to less than 1 hour |
| **6.** | 1 hour or more |

**Regarding prospects and treatment strategies (continuation of current treatment, change to maintenance therapy, transition to observation without treatment, etc.)**

**Since your condition began to stabilize**, have you had the opportunity to speak with your physician again about your prospects and treatment strategies, including their explanations and any questions you may have had?

(Select one)

| **1.** | Yes, **I have had** the opportunity to speak again about prospects and treatment strategies  **→ Go to next page ‎Q18** |
| --- | --- |
| **2.** | No, **I have not had** the opportunity to speak again about prospects or treatment strategies |

**If you have not had the opportunity to discuss prospects or treatment strategies, please answer ‎Q17 below.**


**Since your condition began to stabilize**, would you like the opportunity to speak with your physician again about your prospects and treatment strategies, including their explanations and any questions you may have?

(Select one)

| I would like that very much | I would like that | Not sure | I would not like that very much | I would not like that at all |
| --- | --- | --- | --- | --- |
| **5** | **4** | **3** | **2** | **1** |

**↓**

**After answering, please proceed to ‎Q21 on page 19.**

**If** you have had the opportunity to discuss prospects and treatment strategies,
**Please answer questions ‎Q18** to [**Q20**](#link_Q20).


**Since your condition began to stabilize**, when you had the opportunity to speak with your physician again **about your prospects and treatment strategies**, **how much of that time was spent on the physician’s explanation**?

How much **time did you have to ask your physician questions or discuss your concerns**?

*If explanations and opportunities for questions occur multiple times, please provide the total time spent.

|  | **Amount of time  spent listening to the physician’s explanation**  (Select one) | **Amount of time you were able to consult**  **with the physician and ask questions**  (Select one) |
| --- | --- | --- |
| Less than 15 minutes | **1** | **1** |
| 15 minutes to less than 30 minutes | **2** | **2** |
| 30 minutes to less than 1 hour | **3** | **3** |
| 1 hour or more | **4** | **4** |

Regarding the physician’s explanation **when you had the opportunity to speak with them again since your condition began to stabilize**…

(Select all that apply)

|  | **About the disease** |
| --- | --- |
| **1.** | The physician explained multiple myeloma |
| **2.** | The physician explained why treatment is necessary |
|  | **About the treatment options** |
| **3.** | The physician explained maintenance therapy and continuous therapy, as well as the possibility of observation without treatment |
| **4.** | The physician explained the pros and cons of each treatment |
| **5.** | The physician also presented observation without treatment as an option |
| **6.** | The physician asked about my preference |
|  | **About prospects** |
| **7.** | The physician explained maintenance therapy after initiation (duration of treatment, timing and methods used to assess treatment effectiveness, etc.) |
| **8.** | The physician informed me that treatment may be changed depending on the therapeutic effect and that I will be consulted at that time |
|  | **When you receive an explanation from the physician** |
| **9.** | The physician confirmed that I understood their explanation |
| **10.** | The physician listened to my questions, concerns, and worries and responded to them |
| **11.** | The physician encouraged me to ask questions |
| **12.** | The physician used pamphlets, including notes, to explain the information (and gave me a pamphlet) |
|  |  |
| **13.** | None of the above/I don’t remember |


How well did you understand **the explanation** given by the physician **about multiple myeloma when you had the opportunity to talk with them again**?

(Select one)

| Very well | Moderately well | Not sure | Not very well | Not at all |
| --- | --- | --- | --- | --- |
| **5** | **4** | **3** | **2** | **1** |

How well did you understand **the explanation** given by the physician **about treatment options** **when you had the opportunity to talk with them again**?

(Select one)

| Very well | Moderately well | Not sure | Not very well | Not at all |
| --- | --- | --- | --- | --- |
| **5** | **4** | **3** | **2** | **1** |

**Regarding your thoughts on treatment**

**From this point forward, everyone will respond.**


What are your **current expectations for** multiple myeloma **treatment (treatment regimen)** **since your condition began to stabilize**? Please indicate the **degree of importance** you place on your expectations for the treatment (treatment regimen) you receive. Please also provide your **top five priorities**.

|  | Very important | Moderately important | Not sure | Not very important | Not important at all |  | Top five priorities |
| --- | --- | --- | --- | --- | --- | --- | --- |
|  | (Select one for each row) | | | | |  | (Enter number) |
| Longer survival | **5** | **4** | **3** | **2** | **1** |  | position |
| Long time to recurrence | **5** | **4** | **3** | **2** | **1** |  | position |
| Improved quality of life | **5** | **4** | **3** | **2** | **1** |  | position |
| Long-lasting treatment effect | **5** | **4** | **3** | **2** | **1** |  | position |
| Achieve an early response | **5** | **4** | **3** | **2** | **1** |  | position |
| Strong therapeutic effect | **5** | **4** | **3** | **2** | **1** |  | position |
| Fewer adverse reactions | **5** | **4** | **3** | **2** | **1** |  | position |
| Convenient administration methods (intravenous, subcutaneous, oral administration) | **5** | **4** | **3** | **2** | **1** |  | position |
| Fewer hospital visits | **5** | **4** | **3** | **2** | **1** |  | position |
| Less economic burden | **5** | **4** | **3** | **2** | **1** |  | position |

**Since your condition began to stabilize**, what has been your top priority when considering your expectations for multiple myeloma treatment that you mentioned in your answer to Q21? Please select **the most appropriate option:**

(Select one)

| **1.** | Impact on my family |
| --- | --- |
| **2.** | Impact on my work |
| **3.** | Impact on my hobbies |
| **4.** | Impact on social engagement activities |
| **5.** | None of the above |

**Since your condition began to stabilize**, have you been able to share with your physician the treatment expectations and priorities (values) that you mentioned in your answers to Q21 and Q22?

(Select one)

| **1.** | **I have shared** my treatment expectations and priorities (values) with my physician |
| --- | --- |
| **2.** | **I have not shared** my treatment expectations and priorities (values) with my physician |

**Now that your condition is stable**, how would you like to decide on treatment with your physician?

(Select one)

| **1.** | Make all treatment decisions on my own |
| --- | --- |
| **2.** | Make the final treatment decision on my own after carefully considering the physician’s opinion |
| **3.** | Decide with the physician which treatment is best for me |
| **4.** | Leave the final decision to the physician on which treatment to receive, but my thoughts are considered |
| **5.** | Leave all decisions on treatment to the physician |

**Since your condition began to stabilize**, what would you like to discuss or wish you had discussed with your physician (regardless of whether or not you have already done so)?

(Select all that apply)

| **1.** | A desire to change to maintenance therapy |
| --- | --- |
| **2.** | A question about how long the treatment will continue |
| **3.** | A desire to stop treatment for a while |
| **4.** | Other: Please describe below |

**Regarding your knowledge of the disease and your emotions**

**The following questions will address your knowledge and emotions regarding multiple myeloma.**

How much did you know about **multiple myeloma** at treatment initiation?

In addition, now that your condition is stable, how much do you currently know?

**At treatment initiation:** About multiple myeloma

(Select one)

| I had detailed knowledge | I had some knowledge | Neutral | I had very little knowledge | I had no knowledge  at all |
| --- | --- | --- | --- | --- |
| **5** | **4** | **3** | **2** | **1** |

**Now that your condition is stable:** About multiple myeloma

(Select one)

| Detailed knowledge | Some knowledge | Neutral | Very little knowledge | No knowledge at all |
| --- | --- | --- | --- | --- |
| **5** | **4** | **3** | **2** | **1** |

How much did you know about **multiple** **myeloma treatment options** at treatment initiation?

In addition, now that your condition is stable, how much do you currently know?

**At treatment initiation:** About treatment options for multiple myeloma

(Select one)

| I had detailed knowledge | I had some knowledge | Neutral | I had very little knowledge | I had no knowledge  at all |
| --- | --- | --- | --- | --- |
| **5** | **4** | **3** | **2** | **1** |

**Now that your condition is stable:** About treatment options for multiple myeloma

(Select one)

| Detailed knowledge | Some knowledge | Neutral | Very little knowledge | No knowledge  at all |
| --- | --- | --- | --- | --- |
| **5** | **4** | **3** | **2** | **1** |

Please share your thoughts and emotions **both at treatment initiation and at present, now that your condition is stable**.

(Select all that apply)

Thoughts and emotions **at treatment initiation**

| **1.** | Confused | **7.** | Powerless | **13.** | Other: Please describe below |
| --- | --- | --- | --- | --- | --- |
| **2.** | Excited | **8.** | Sad |  | ( ) |
| **3.** | Frustrated | **9.** | Disappointed |  | ( ) |
| **4.** | Optimistic/Hopeful | **10.** | Guilty |  |  |
| **5.** | Fearful | **11.** | Apologetic |  |  |
| **6.** | Confident | **12.** | Worried |  |  |

Thoughts and emotions **at present, while in a stable condition**

| **1.** | Confused | **7.** | Powerless | **13.** | Other: Please describe below |
| --- | --- | --- | --- | --- | --- |
| **2.** | Excited | **8.** | Sad |  | ( ) |
| **3.** | Sense of failure | **9.** | Disappointed |  | ( ) |
| **4.** | Optimistic/ Hopeful | **10.** | Guilty |  |  |
| **5.** | Fearful | **11.** | Apologetic |  |  |
| **6.** | Confident | **12.** | Worried |  |  |

**Information about your disease**

**We will now ask questions about how you gather information on multiple myeloma.**

Where do you usually learn information about multiple myeloma and its treatment?
Additionally, which pieces of information specifically influence your treatment decision?

|  | **Your usual source of  information**  (Select all that apply) | Which pieces of **information**  **specifically influence your treatment decision?**  (Select all that apply) |
| --- | --- | --- |
| Physician whom you regularly visit for a medical examination | **1** | **1** |
| Nurse | **2** | **2** |
| Pharmacist | **3** | **3** |
| Hematologist-Oncologist (for a second opinion) other than your usual physician | **4** | **4** |
| Primary care physician whom you visit for other conditions | **5** | **5** |
| Pharmaceutical company website | **6** | **6** |
| Website other than pharmaceutical company | **7** | **7** |
| Social media (e.g. Facebook) | **8** | **8** |
| Online videos (e.g. YouTube) | **9** | **9** |
| Other patients’ blogs and bulletin boards | **10** | **10** |
| Nihon Kotsuzuishu Kanja No Kai (Myeloma Patients and Families, Japan) (e.g. Ganbarimasshoi, seminars, etc.) | **11** | **11** |
| Patient organizations other than Nihon Kotsuzuishu Kanja No Kai (Myeloma Patients and Families, Japan) | **12** | **12** |
| Public lectures | **13** | **13** |
| Friends and family | **14** | **14** |
| Magazines and books | **15** | **15** |
| None of the above | **16** | **16** |

**Finally, we will ask you some questions about yourself.**

**All data will be collected and analyzed anonymously, and will remain confidential.**

How old are you?

(Enter number)

**( ) years old**

Please indicate your gender.

(Select one)

| **1.** | Male |
| --- | --- |
| **2.** | Female |
| **3.** | Other |
| **4.** | Prefer not to answer |

Please select the region where you live.

(Select one)

| **1.** | Hokkaido/Tohoku |
| --- | --- |
| **2.** | Kanto |
| **3.** | Chubu |
| **4.** | Kinki |
| **5.** | Chugoku/Shikoku |
| **6.** | Kyushu/Okinawa |
| **7.** | Prefer not to answer |

Please provide your current employment status.

(Select one)

| **1.** | Currently employed |
| --- | --- |
| **2.** | Currently unemployed |
| **3.** | Prefer not to answer |

Tell us about your lifestyle.

(Select one)

| **1.** | Living with family |
| --- | --- |
| **2.** | Living alone |
| **3.** | Prefer not to answer |

**The survey is now complete. Thank you for your responses.**

**Please use the enclosed envelope to return the questionnaire to us by Wednesday, November 6, 2024.**

**Supplemental Text 2 |** English translation of the physician questionnaire.

**Physician Questionnaire**

**Questionnaire About Treatment of Blood Diseases**

| [Conditions for respondents]   - Hematologist - There are several other conditions   [Scope of disclosure, etc.] In accordance with the Act on the Protection of Personal Information and its guidelines, only aggregated results that do not identify individual respondents will be disclosed to the following extent (■):  □ 1. Will be disclosed to our company and the survey sponsor  □ 2. May be disclosed to healthcare professionals under confidentiality agreements and relevant agencies for the purpose of advisory services to the survey sponsor  □ 3. May be disclosed to members of m3.com (or on the m3.com website)  □ 4. May be disclosed only to medical personnel  ■ 5. May be disclosed to the public  In addition, we may process questionnaire respondents as necessary, as described below, and share individual responses, aggregated results, and analysis results with third parties, to the extent that individual respondents cannot be identified.  • The respondents of this questionnaire are matched to the relevant classification based on the classification provided by the company or a third party.  • The respondents of this questionnaire are matched to the results of previous related questionnaires.  [Response method]  If you close your browser before the survey deadline, your completed responses will be automatically saved. When you return, you can resume from where you left off. |
| --- |

Now select the following and click “Next” to continue.

○Doctor of Hematology

Next

---Page change---

What is your specialty?

(Select one)

| Hematologist | 1 |
| --- | --- |
| Oncologist | 2 |
| Other than above (general internist, etc.) | 3 |

---

Screen-out for non-hematologists

How many patients with blood disorders have you seen in the past year based on patient charts?

(Enter number)

**Please indicate your main place of work.**

| Acute myeloid leukemia | patients/year |
| --- | --- |
| Chronic myeloid leukemia | patients/year |
| Malignant lymphoma | patients/year |
| Multiple myeloma | patients/year |
| Myelodysplastic syndrome | patients/year |

---

Screen-out if less than 2 multiple myeloma patients

[Sponsor] Takeda Pharmaceutical Company Limited

[Research company] Social Survey Research Information Co., Ltd.

**Real-World Survey on the Treatment of** **Multiple Myeloma**

The survey will include a letter of invitation and questions and will take approximately 20 minutes to complete.

[Precautions]

If you understand the following precautions and would like to cooperate with the survey, please select “I agree” below.

• Purpose of this survey: This survey aims to assess the current state of and opinions on treatment selection among doctors currently treating patients with multiple myeloma.

• Please answer each question by selecting the options that apply to your situation. Some of the questions may ask you to enter a number.

• Your participation in this survey is not mandatory. Your decision to answer is voluntary.

• If you wish to stop answering midway, you may do so immediately.

• Failure to respond or withdrawal from the survey will not disadvantage you in any way.

• Responses remain confidential throughout processing, storage, and disposal. Your personal information will not be disclosed.

• The results of this survey will be used solely for research purposes, processed statistically to ensure anonymity, and presented at academic conferences or published as a scholarly paper. The results will not be published in a way that reveals individual identities.

• This survey is being conducted by the research company contracted by M3, Inc. at the request of the sponsor. All costs related to the survey will be paid for by the sponsor. The ethics committee has reviewed and confirmed in advance that no conflict of interest exists between the sponsor and the research company that could compromise the reliability of the survey, and that the appropriate implementation structure is in place.

• Intellectual property rights arising from this survey shall belong to the sponsor.

• The results of the survey may be provided to regulatory authorities and ethics committees as necessary, but individuals will remain anonymous in all cases.

The survey sponsor has requested that if any adverse reactions or harmful events related to their products arise, the details should be reported.

The purpose of this use is limited to adverse event reporting. However, we will confirm your preferences regarding the handling of adverse event names and personal information and take appropriate measures to ensure you are not inconvenienced in any way.

After selecting “I agree”, please continue to the next screen and answer the questions.

I agree ○ I do not agree ○

Please proceed to the next screen.

**We would now like to ask about the circumstances under which treatment is initiated for patients diagnosed with multiple myeloma who are not eligible for hematopoietic stem cell transplantation.**

**From diagnosis to treatment initiation,** how much time do you set aside to provide an explanation (of diseases and treatment options) to patients **not eligible for hematopoietic stem cell transplantation**?

In addition, how much time do you allocate for patients to ask questions and consult with you?

*Please indicate the most common cases of multiple myeloma you have treated.

*If explanations and opportunities for questions occur multiple times, please provide the total time spent.

|  | Time spent explaining diseases and treatment options to patients  (Select one from below) | Time available for patients to ask questions and consult with you  (Select one from below) |
| --- | --- | --- |
| Less than 15 minutes | 1 | 1 |
| 15 minutes to less than 30 minutes | 2 | 2 |
| 30 minutes to less than 1 hour | 3 | 3 |
| 1 hour or more | 4 | 4 |

Please describe everything that you usually do during the consultation **from diagnosis to treatment** **initiation** for patients **not eligible for hematopoietic stem cell transplantation**.

*Please indicate the most common cases of multiple myeloma you have treated.

(Select all that apply)

| **About the disease** |  |
| --- | --- |
| Explanation about multiple myeloma | 1 |
| Explanation about why treatment is necessary | 2 |
| **About the treatment options** |  |
| Presentation of several treatment options | 3 |
| Explanation about the pros and cons of each treatment | 4 |
| Explanation that observation without treatment is an option | 5 |
| Ask about patient preference | 6 |
| **About the patient’s prospects after treatment initiation** |  |
| Explanation about the patient’s prospects after treatment initiation (duration of treatment, timing and methods used to assess treatment effectiveness, etc.) | 7 |
| Inform the patient that treatment may be changed depending on the therapeutic effect and that they will be consulted at that time | 8 |
| **How the consultation is conducted** |  |
| Confirm if the patient understands the physician’s explanation | 9 |
| Listen to questions, concerns, and worries from the patient, and respond to them | 10 |
| Encourage the patient to ask questions | 11 |
| Give an explanation using pamphlets, including notes (a pamphlet was provided to the patient) | 12 |
|  |  |
| None of the above | 13 |

What are your expectations for a multiple myeloma treatment regimen at **treatment initiation for patients who are not eligible for hematopoietic stem cell transplantation**? Please indicate the degree of importance you place on the desired aspects of the treatment regimen.

Please also provide your top five priorities.

*Please indicate the most common cases of multiple myeloma you have treated.

|  | Very important | Moderately important | Not sure | Not very important | Not important at all |  | Top five priorities |
| --- | --- | --- | --- | --- | --- | --- | --- |
|  | (Select one for each row) | | | | |  | (Enter number) |
| Longer survival | 1 | 2 | 3 | 4 | 5 |  | position |
| Long time to recurrence | 1 | 2 | 3 | 4 | 5 |  | position |
| Improved quality of life | 1 | 2 | 3 | 4 | 5 |  | position |
| Long-lasting treatment effect | 1 | 2 | 3 | 4 | 5 |  | position |
| Achieve an early response | 1 | 2 | 3 | 4 | 5 |  | position |
| Strong therapeutic effect | 1 | 2 | 3 | 4 | 5 |  | position |
| Fewer adverse reactions | 1 | 2 | 3 | 4 | 5 |  | position |
| Convenient administration methods (intravenous, subcutaneous, oral administration) | 1 | 2 | 3 | 4 | 5 |  | position |
| Fewer hospital visits | 1 | 2 | 3 | 4 | 5 |  | position |
| Less economic burden | 1 | 2 | 3 | 4 | 5 |  | position |

**At treatment initiation for patients not eligible for hematopoietic stem cell transplantation,**

with how many of your patients are you able to discuss their priorities and values regarding their desired treatment options?

Examples of patients’ priorities (values) include:

Impact on family, impact on work, impact on hobbies, impact on social engagement activities, etc.

(Select one)

| With 80% or more of patients | 1 |
| --- | --- |
| With 60% or more to less than 80% of patients | 2 |
| With 40% or more to less than 60% of patients | 3 |
| With 20% or more to less than 40% of patients | 4 |
| With less than 20% of patients | 5 |

**When initiating treatment for patients not eligible for hematopoietic stem cell transplantation**, how do you think they prefer to make treatment decisions?

*Please indicate the most common cases of multiple myeloma you have treated.

(Select one)

| They make all treatment decisions on their own | 1 |
| --- | --- |
| They make the final treatment decision on their own after carefully considering the physician’s opinion | 2 |
| They decide with a physician which treatment is best for them | 3 |
| They leave the final decision to a physician on which treatment to receive, but their thoughts are considered | 4 |
| They leave all decisions on treatment to the physician | 5 |

**We would now like to ask about the condition of patients who are not eligible for hematopoietic stem cell transplantation** **after they have stabilized following induction therapy.**

Regarding **patients in a stable disease phase following induction therapy**:

How much time do you spend on a single consultation? Please indicate the average duration of consultations in your regular clinical practice.

*Please indicate the most common cases of multiple myeloma you have treated.

(Select one)

| Less than 5 minutes | 1 |
| --- | --- |
| 5 minutes to less than 10 minutes | 2 |
| 10 minutes to less than 20 minutes | 3 |
| 20 minutes to less than 30 minutes | 4 |
| 30 minutes to less than 1 hour | 5 |
| 1 hour or more | 6 |

---

Regarding **patients in a stable disease phase following induction therapy:**

How often do you have the opportunity to have follow-up discussions on prospects and treatment strategies with the patient, including explanations from the physician, patient questions, and consultations?

(Select one)

| I provide 80% or more of the patients with an opportunity for a follow-up discussion | 1 |
| --- | --- |
| I provide 60% or more and less than 80% of the patients with an opportunity for a follow-up discussion | 2 |
| I provide 40% or more and less than 60% of the patients with an opportunity for a follow-up discussion | 3 |
| I provide 20% or more and less than 40% of the patients with an opportunity for a follow-up discussion | 4 |
| I provide 20% or less of the patients with an opportunity for a follow-up discussion | 5 |

Regarding **patients in a stable disease phase following induction therapy:**

Please provide the reason for not offering the patient an opportunity to discuss their prospects and treatment strategies again.

(Select all that apply)

| Because the patient’s condition is stable | 1 |
| --- | --- |
| Because there is no alternative treatment available, even after further discussion | 2 |
| Because there is no time | 3 |
| Because there are no questions or requests from patients | 4 |
| None of the above | 5 |

Regarding **patients in a stable disease phase following induction therapy**:

How much time do you spend in follow-up discussions with the patient (including explanations from the physician, patient questions, and consultations)?

In addition, how much time do you allocate for patients to ask questions and consult with you?

*Please indicate the most common cases of multiple myeloma you have treated.

*If explanations and opportunities for questions occur multiple times, please provide the total time spent.

|  | Time spent explaining diseases and treatment options to patients  (Select one from below) | Time available for patients to ask questions and consult with you  (Select one from below) |
| --- | --- | --- |
| Less than 15 minutes | 1 | 1 |
| 15 minutes to less than 30 minutes | 2 | 2 |
| 30 minutes to less than 1 hour | 3 | 3 |
| 1 hour or more | 4 | 4 |

Regarding **patients in a stable disease phase following induction therapy**:

During your opportunity to speak with patients again (including explanations from the physician, patient questions, and consultations), please describe everything you typically do.

*Please indicate the most common cases of multiple myeloma you have treated.

(Select all that apply)

| **About the disease** |  |
| --- | --- |
| Explanation about multiple myeloma | 1 |
| Explanation about why treatment is necessary | 2 |
| **About the treatment** |  |
| Explanation about treatment options of maintenance therapy,  continuous therapy, and observation without treatment | 3 |
| Explanation about the pros and cons of each treatment | 4 |
| Explanation that observation without treatment is an option | 5 |
| Ask about patient preference | 6 |
| **About prospects** |  |
| Explanation about maintenance therapy after initiation, including treatment duration, timing and methods used to assess treatment effectiveness, etc. | 7 |
| Inform the patient that treatment may be changed depending on the therapeutic effect and that they will be consulted at that time | 8 |
| **How the consultation is conducted** |  |
| Confirm if the patient understands the physician’s explanation | 9 |
| Listen to questions, concerns, and worries from the patient, and respond to them | 10 |
| Encourage the patient to ask questions | 11 |
| Give an explanation using pamphlets, including notes (a pamphlet was provided to the patient) | 12 |
|  |  |
| None of the above | 13 |

Regarding **patients who have been treated with induction therapy and are in a stable disease phase,** what are your expectations for a multiple myeloma treatment regimen? Please indicate the degree of importance you place on the desired aspects of the treatment regimen.

Please also provide your top five priorities.

*Please indicate the most common cases of multiple myeloma you have treated.

|  | Very important | Moderately important | Not sure | Not very important | Not important at all |  | Top five priorities |
| --- | --- | --- | --- | --- | --- | --- | --- |
|  | (Select one for each row) | | | | |  | (Enter number) |
| Prolonged survival | 1 | 2 | 3 | 4 | 5 |  | position |
| Long time to recurrence | 1 | 2 | 3 | 4 | 5 |  | position |
| Improved quality of life | 1 | 2 | 3 | 4 | 5 |  | position |
| Long-lasting treatment effect | 1 | 2 | 3 | 4 | 5 |  | position |
| Achieve an early response | 1 | 2 | 3 | 4 | 5 |  | position |
| Strong therapeutic effect | 1 | 2 | 3 | 4 | 5 |  | position |
| Fewer adverse reactions | 1 | 2 | 3 | 4 | 5 |  | position |
| Convenient administration methods (intravenous, subcutaneous, oral administration) | 1 | 2 | 3 | 4 | 5 |  | position |
| Fewer hospital visits | 1 | 2 | 3 | 4 | 5 |  | position |
| Less economic burden | 1 | 2 | 3 | 4 | 5 |  | position |

Regarding **patients who have been treated with induction therapy and are in a stable disease phase,** with how many of your patients are you able to discuss their priorities and values regarding their desired treatment options?

Examples of patients’ priorities (values) include:

Impact on family, impact on work, impact on hobbies, impact on social engagement activities, etc.

(Select one)

| With 80% or more of patients | 1 |
| --- | --- |
| With 60% or more to less than 80% of patients | ２ |
| With 40% or more to less than 60% of patients | 3 |
| With 20% or more to less than 40% of patients | 4 |
| With less than 20% of patients | 5 |

What treatment decisions do you think **patients would like to make during periods of stable disease following induction therapy?**

*Please indicate the most common cases of multiple myeloma you have treated.

(Select one)

| They make all treatment decisions on their own | 1 |
| --- | --- |
| They make the final treatment decision on their own after carefully considering the physician’s opinion | 2 |
| They decide with a physician which treatment is best for them | 3 |
| They leave the final decision to a physician on which treatment to receive, but their thoughts are considered | 4 |
| They leave all decisions on treatment to the physician | 5 |

Please describe what **patients in stable condition following induction therapy** typically communicate to you.

|  | More than 80% of patients communicate to me… | 60% or more to less than 80% of patients communicate to me… | 40% or more to less than 60% of patients communicate to me… | 20% or more to less than 40% of patients communicate to me… | Less than 20% of patients communicate to me… |
| --- | --- | --- | --- | --- | --- |
|  | (Select one for each row) | | | | |
| A desire to change to maintenance therapy | 1 | 2 | 3 | 4 | 5 |
| A question about how long the treatment will continue | 1 | 2 | 3 | 4 | 5 |
| A desire to stop treatment for a while | 1 | 2 | 3 | 4 | 5 |

**Finally, please specify your main place of work for statistical analysis of your responses.**

| **Face Sheet (Demographic Profile Questions)** | |
| --- | --- |
| F1. Facility management form  (Select one) | 1. Cancer Center/Academia  2. Community hospital (national/government hospital, public hospital or general hospital other than the above), Medical office/Clinic  3. Other |
| F2. Total number of beds at site  (Select one) | 1. 0 beds  2. 1–19 beds  3. 20–99 beds  4. 100–199 beds  5. 200–499 beds  6. 500 beds or more |
| F3. Region  (Select one) | 1. Hokkaido/Tohoku  2. Kanto  3. Chubu  4. Kinki  5. Chugoku/Shikoku  6. Kyushu/Okinawa |
| F4. Doctor’s age  (Select one) | 1. 20s  2. 30s  3. 40s  4. 50s  5. 60s  6. 70 years or older |

End of document

**Table S1** | Time spent during medical consultation reported by patients and physicians at treatment initiation and disease stabilization.

|  | **Patients**  **(*N*=220)** | **Physicians**  **(*N*=120)** |
| --- | --- | --- |
| **Treatment initiation** |  |  |
| Time spent on explanation about the disease and treatment options, min | | |
| <15 | 38 (17.3) | 12 (10.0) |
| 15 to <30 | 72 (32.7) | 56 (46.7) |
| 30 to <60 | 72 (32.7) | 43 (35.8) |
| ≥60 | 36 (16.4) | 9 (7.5) |
| Unknown | 2 (0.9) | 0 (0) |
| Time spent on questions and discussion, min | | |
| <15 | 81 (36.8) | 47 (39.2) |
| 15 to <30 | 64 (29.1) | 58 (48.3) |
| 30 to <60 | 41 (18.6) | 10 (8.3) |
| ≥60 | 28 (12.7) | 5 (4.2) |
| Unknown | 6 (2.7) | 0 (0) |
| **Disease stabilization** |  |  |
| Mean time spent on medical consultation, min | | |
| <5 | 39 (17.7) | 10 (8.3) |
| 5 to ≤10 | 105 (47.7) | 67 (55.8) |
| 10 to ≤20 | 51 (23.2) | 27 (22.5) |
| 20 to ≤30 | 12 (5.5) | 7 (5.8) |
| 30 to ≤60 | 2 (0.9) | 5 (4.2) |
| ≥60 | 8 (3.6) | 4 (3.3) |
| Unknown | 3 (1.4) | 0 (0) |
| Time spent on explanation about the disease and treatment options, min | | |
| *n* | 163^a^ | 120 |
| <15 | 85 (52.1) | 69 (57.5) |
| 15 to <30 | 50 (30.7) | 31 (25.8) |
| 30 to <60 | 13 (8.0) | 14 (11.7) |
| ≥60 | 11 (6.7) | 6 (5.0) |
| Unknown | 4 (2.5) | 0 (0) |
| Time spent on questions and discussion, min | | |
| *n* | 163^a^ | 120 |
| <15 | 99 (60.7) | 77 (64.2) |
| 15 to <30 | 41 (25.2) | 31 (25.8) |
| 30 to <60 | 10 (6.1) | 8 (6.7) |
| ≥60 | 8 (4.9) | 4 (3.3) |
| Unknown | 5 (3.1) | 0 (0) |

^a^Patients who responded that they had an opportunity to discuss prospects and treatment strategies at disease stabilization.

Abbreviation: min, minutes.

**Table S2** | Reasons why physicians did not provide opportunities to discuss prospects and treatment strategies at disease stabilization.

|  | **Physicians (*N*=120)** |
| --- | --- |
| Patient’s condition is stable | 75 (62.5) |
| There are no questions or requests from the patient | 41 (34.2) |
| There is/are no alternative treatment/s | 39 (32.5) |
| There is no time for discussion | 31 (25.8) |
| None of the above | 8 (6.7) |
| Unknown | 0 (0) |

**Table S3** | Topics that patients hoped to discuss with the physician at disease stabilization.

|  | **Patients (*N*=220)** |
| --- | --- |
| Duration of treatment | 139 (63.2) |
| Changing treatment to maintenance therapy | 31 (14.1) |
| Stopping treatment for a while | 15 (6.8) |
| Other | 63 (28.6) |
| Unknown | 19 (8.6) |

**Figure S1** | Physicians’ treatment expectations at treatment initiation and disease stabilization. Physicians rated the importance of each treatment attribute on a scale of “not important at all” to “very important”; the figure shows the proportion of physicians who rated “very important” or “moderately important”. QoL, quality of life.


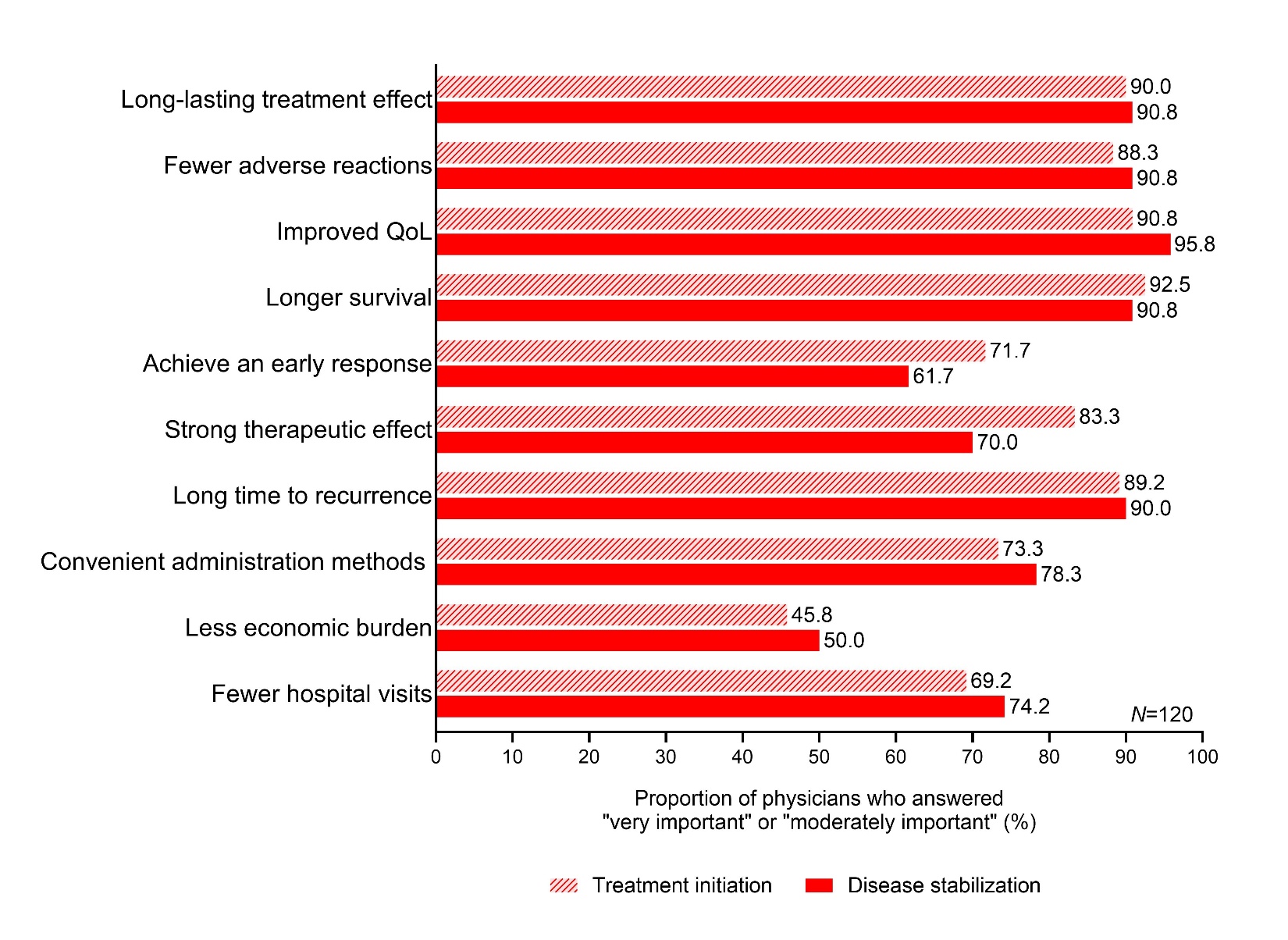


**Figure S2** | Patients’ level of understanding of **(A)** the disease, **(B)** treatment options at treatment initiation and disease stabilization. Patients answered about their level of understanding. ^a^*p*-Values calculated by Wilcoxon signed rank test show the overall change in patients’ level of understanding at treatment initiation versus disease stabilization. ^b^Patients who responded that they had an opportunity to discuss prospects and treatment strategies at disease stabilization.


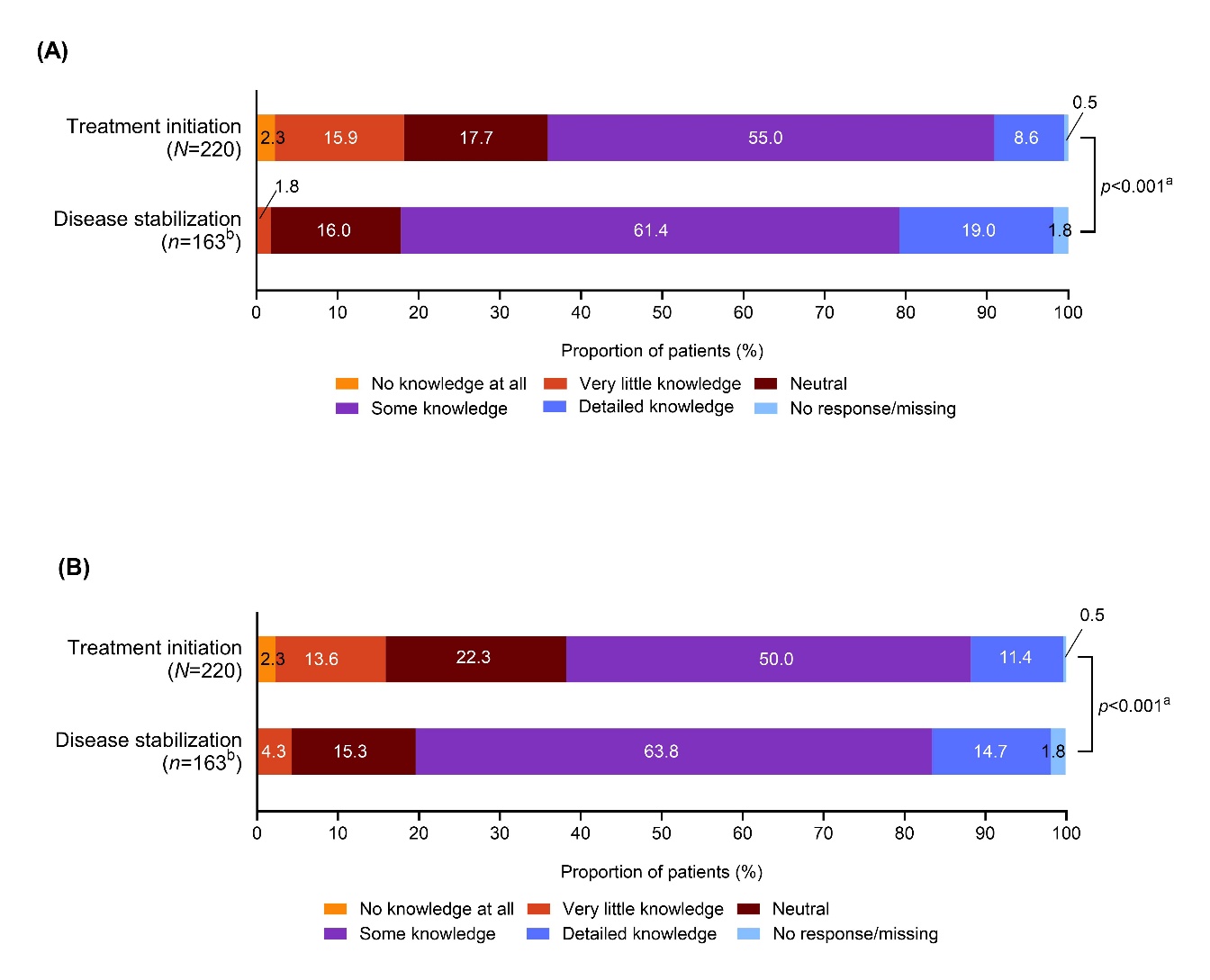


**Figure S3** | Patients’ sources of information on the disease and treatment.


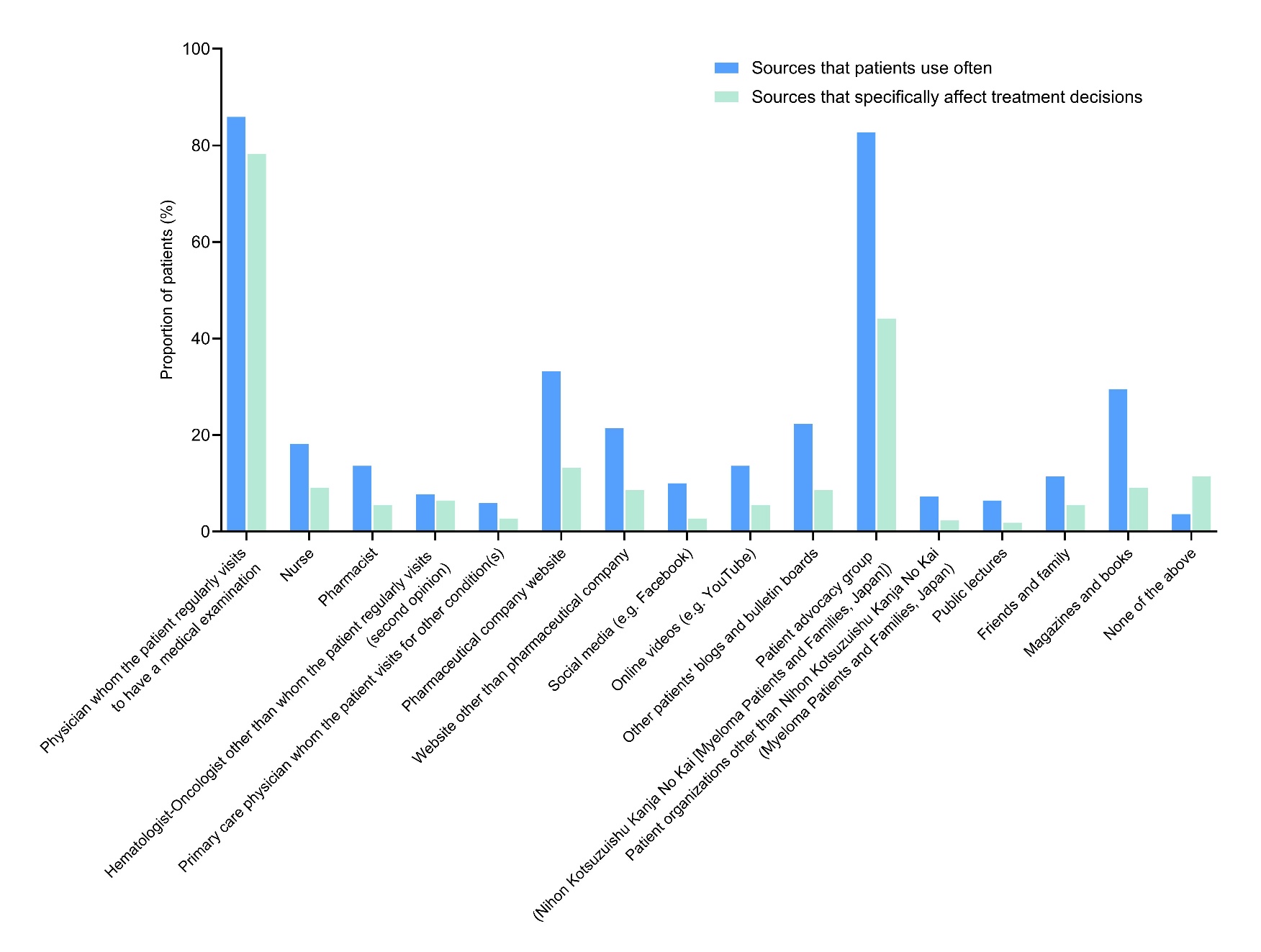


**Figure S4** | Physicians’ perceptions: patients’ preferred treatment decision-making role at treatment initiation and disease stabilization. Physicians answered about their perception of the patients’ preferred roles at treatment initiation and disease stabilization.


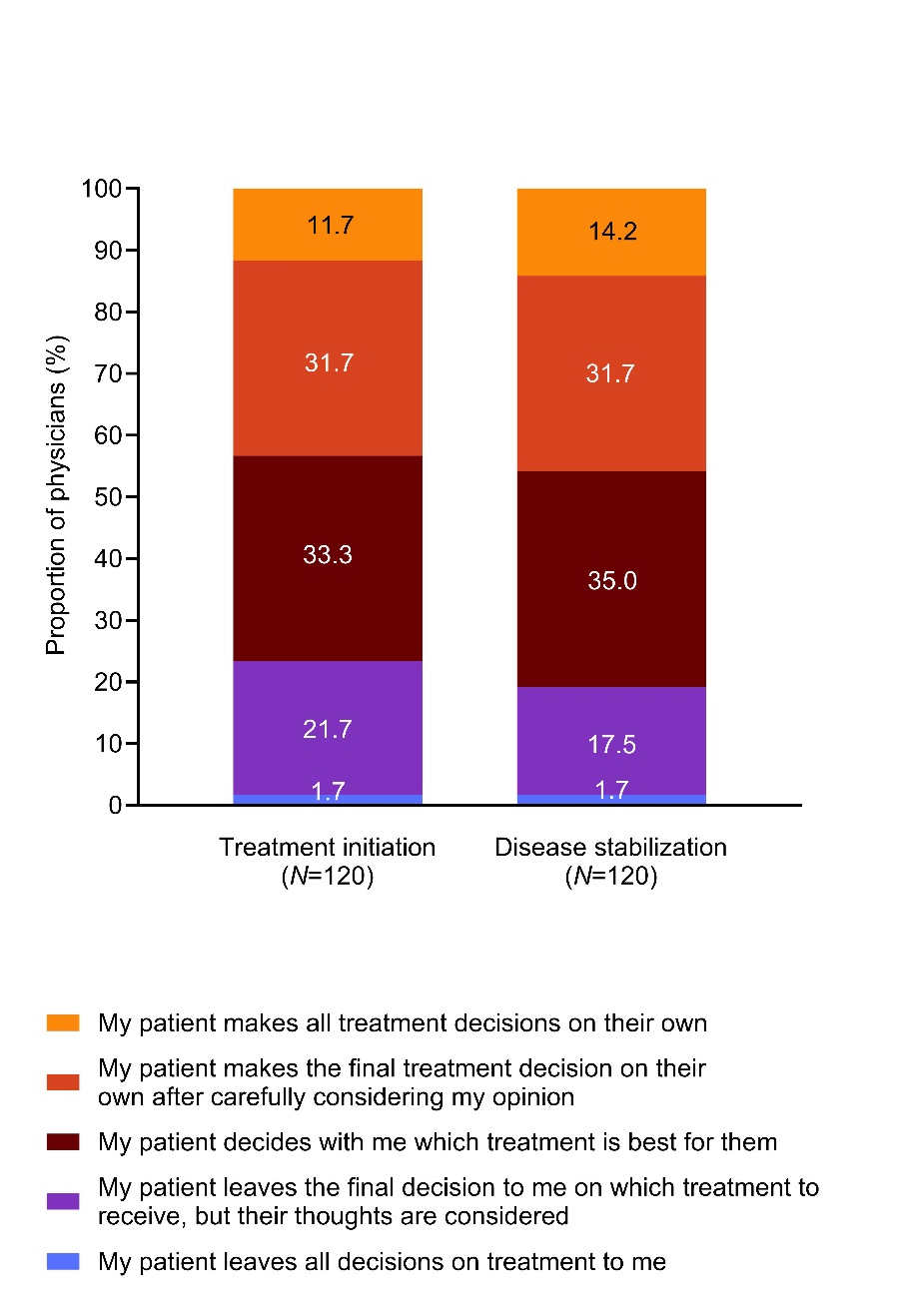

Supplement: Supplementary file 1 — Supporting File 1: jha270247‐sup‐0001‐SuppMat.docx [file JHA2-7-e70247-s001.docx]
